# Acoustic features as a tool to visualize and explore marine soundscapes: Applications illustrated using marine mammal passive acoustic monitoring datasets

**DOI:** 10.1002/ece3.10951

**Published:** 2024-02-21

**Authors:** Simone Cominelli, Nicolo' Bellin, Carissa D. Brown, Valeria Rossi, Jack Lawson

**Affiliations:** ^1^ Northern EDGE Lab, Department of Geography Memorial University of Newfoundland and Labrador St. John's Newfoundland and Labrador Canada; ^2^ Department of Chemistry, Life Sciences and Environmental Sustainability University of Parma Parma Italy; ^3^ Marine Mammal Section Department of Fisheries and Oceans St. John's Newfoundland and Labrador Canada

**Keywords:** ecoacoustics, machine learning, marine mammals, passive acoustic monitoring, UMAP

## Abstract

Passive Acoustic Monitoring (PAM) is emerging as a solution for monitoring species and environmental change over large spatial and temporal scales. However, drawing rigorous conclusions based on acoustic recordings is challenging, as there is no consensus over which approaches are best suited for characterizing marine acoustic environments. Here, we describe the application of multiple machine‐learning techniques to the analysis of two PAM datasets. We combine pre‐trained acoustic classification models (VGGish, NOAA and Google Humpback Whale Detector), dimensionality reduction (UMAP), and balanced random forest algorithms to demonstrate how machine‐learned acoustic features capture different aspects of the marine acoustic environment. The UMAP dimensions derived from VGGish acoustic features exhibited good performance in separating marine mammal vocalizations according to species and locations. RF models trained on the acoustic features performed well for labeled sounds in the 8 kHz range; however, low‐ and high‐frequency sounds could not be classified using this approach. The workflow presented here shows how acoustic feature extraction, visualization, and analysis allow establishing a link between ecologically relevant information and PAM recordings at multiple scales, ranging from large‐scale changes in the environment (i.e., changes in wind speed) to the identification of marine mammal species.

## Graphical abstract

Our study explores the use of VGGish acoustic features and UMAP dimensionality reduction for the analysis of marine soundscapes. We combine pre‐trained acoustic classification models, dimensionality reduction (UMAP), and balanced random forest algorithms to demonstrate how machine‐learned acoustic features capture different aspects of the marine environment.

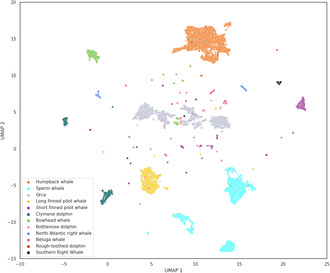

## 1 INTRODUCTION

Abrupt changes in the ocean environment are increasing in frequency as climate change accelerates (Ainsworth et al., 2020), resulting in the loss of key ecosystems (Sully et al., 2019), and shifts in endangered species' distributions (Plourde et al., 2019). Detecting such changes requires both historical and real‐time (or near‐real‐time) data made readily available to managers and decision‐makers. Scientists and practitioners are being tasked with finding efficient solutions for monitoring environmental health and detecting incipient change (Gibb et al., 2019; Kowarski & Moors‐Murphy, 2021). This challenge includes monitoring for changes in species' presence, abundance, distribution, and behavior (Durette‐Morin et al., 2019; Fleming et al., 2018; Root‐Gutteridge et al., 2018), monitoring anthropogenic activity and disturbance levels (Gómez et al., 2018), monitoring changes in the environment (Almeira & Guecha, 2019), detecting harmful events (Rycyk et al., 2020), among others.

Environmental sounds provide a proxy to investigate ecological processes (Gibb et al., 2019; Rycyk et al., 2020), including exploring complex interactions between anthropogenic activity and biota (Erbe et al., 2019; Kunc et al., 2016). Sound provides useful information on environmental conditions and ecosystem health, allowing, for example, the rapid identification of disturbed coral reefs (Elise et al., 2019). In concert, numerous species (i.e., birds, mammals, fish, and invertebrates) rely on acoustic communication for foraging, mating and reproduction, habitat use and other ecological functions (Eftestøl et al., 2019; Kunc & Schmidt, 2019; Luo et al., 2015; Schmidt et al., 2014). The noise produced by anthropogenic activities (e.g., vehicles, stationary machinery, explosions) can interfere with such processes, affecting the health and reproductive success of multiple marine taxa (Kunc & Schmidt, 2019). In response to concerns about noise pollution, increasing effort is being invested in developing, testing, and implementing noise management measures in both terrestrial and marine environments. Consequently, Passive Acoustic Monitoring (PAM) has become a mainstream tool in biological monitoring (Gibb et al., 2019). PAM represents a set of techniques that are used for the systematic collection of acoustic recordings for environmental monitoring. It allows collection of large amounts of acoustic recordings that can then be used to understand changes happening in the environment at multiple spatial and temporal scales.

One of PAM's most common applications is in marine mammal monitoring and conservation. Marine mammals produce complex vocalizations that are species‐specific (if not individually unique), and such vocalizations can be used when estimating species' distributions and habitat use (Durette‐Morin et al., 2019; Kowarski & Moors‐Murphy, 2021). PAM applications in marine mammal research span from the study of their vocalizations and behaviors (Madhusudhana et al., 2019; Vester et al., 2017) to assessing anthropogenic disturbance (Nguyen Hong Duc et al., 2021). PAM datasets can reach considerable sizes, particularly when recorded at high sampling rates, and projects often rely on experts to manually inspect the acoustic recordings for the identification of sounds of interest (Nguyen Hong Duc et al., 2021). For projects involving recordings collected over multiple months at different locations, conducting a manual analysis of the entire dataset can be prohibitive, and often only a relatively small portion of the acoustic recordings is subsampled for analysis.

At its core, studying acoustic environments is a signal detection and classification problem in which a large number of spatially and temporally overlapping acoustic energy sources need to be differentiated to better understand their relative contributions to the soundscape. Such an analytical process, termed acoustic scene classification (Geiger et al., 2013), is a key step in analyzing environmental information collected by PAM recorders. Acoustic scenes can contain multiple overlapping sound sources, which generate complex combinations of acoustic events (Geiger et al., 2013). This definition overlaps with the ecoacoustics definition of soundscape (Farina & Gage, 2017), providing a bridge between the two fields, where a soundscape represents the total acoustic energy contained within an environment and consists of three intersecting sound sources: geological (i.e., geophony), biological (i.e., biophony), and anthropogenic (i.e., anthrophony). A goal of ecoacoustics is to understand how these sources interact and influence each other, with a particular focus on biological‐anthropogenic acoustic interactions. The concept of soundscape has recently been reframed and expanded to encompass three distinct categories: the distal soundscape, the proximal soundscape, and the perceptual soundscape (Grinfeder et al., 2022). The distal soundscape describes the spatial and temporal variation of acoustic signals within a defined area or environment. The proximal soundscape represents acoustic signals that occur at a specific location within a defined area – a distal soundscape can be interpreted as a collection of proximal soundscapes and includes all potential receiver positions. The perceptual soundscape is the subjective interpretation of a specific proximal soundscape and involves sensory and cognitive processes of the individual. In this study, we focus on the analysis of distal soundscapes, which allow investigating how biotic and abiotic factors relate to acoustic recordings.

Automated acoustic analysis can overcome some of the limitations encountered in manual PAM analysis, allowing ecoacoustics researchers to explore full datasets (Houegnigan et al., 2017). Deep learning represents a novel set of computer‐based artificial intelligence approaches, which has profoundly changed biology and ecology research (Christin et al., 2019). Among the deep learning approaches, Convolutional Neural Networks (CNNs) have demonstrated high accuracy in performing image classification tasks, including the classification of spectrograms (i.e., visual representations of sound intensity across time and frequency) (Hershey et al., 2017; LeBien et al., 2020; Stowell, 2022).

CNNs have been applied successfully to several ecological problems, and their use in ecology has been growing (Christin et al., 2019), such as to process camera trap images to identify species, age classes, numbers of animals, and to classify behavior patterns (Lumini et al., 2019; Norouzzadeh et al., 2018; Tabak et al., 2019). CNN's algorithms perform well for acoustic classification (Hershey et al., 2017), including the identification of a growing number of species vocalizations such as crickets and cicadas (Dong et al., 2018), birds and frogs (LeBien et al., 2020), fish (Mishachandar & Vairamuthu, 2021), and lately marine mammals (Usman et al., 2020). Recent applications of deep learning to the study of marine soundscapes include automated detectors for killer whales (Bergler et al., 2019) and humpback whales (Allen et al., 2021), the detection of North Atlantic right whales under changing environmental conditions (Vickers et al., 2021), and the detection of echolocation click trains produced by toothed whales (Roch et al., 2021).

Most CNN applications focus on species detection rather than a broader characterization of the acoustic environment. Furthermore, automated acoustic analysis algorithms often rely on supervised classification based on large datasets of known sounds (i.e., training datasets) used to train acoustic classifiers; creating training datasets is time‐consuming and requires expert‐driven manual classification of the acoustic data (Bittle & Duncan, 2013).

Recent developments in acoustic scene analysis demonstrate how the implementation of acoustic feature sets derived from CNNs, along with the use of dimensionality reduction (UMAP), can improve our ability to understand ecoacoustics datasets while providing a common ground for analyzing recordings collected across multiple environments and temporal scales (Clink & Klinck, 2021; Mishachandar & Vairamuthu, 2021; Sethi et al., 2020).

In this study, we applied multiple machine‐learning techniques to the analysis of two PAM datasets (the Watkins Marine Mammal Sounds Database,^1^ and 2 months of continuous PAM recordings collected by Fisheries and Oceans Canada in Placentia Bay Newfoundland, Canada during July and August 2019, Figure 1). We combined pre‐trained acoustic classification models (VGGish, NOAA and Google Humpback Whale Detector), dimensionality reduction (UMAP), and balanced random forest algorithms to demonstrate how machine‐learned acoustic features capture different aspects of the marine acoustic environment.

- We used the pre‐trained VGGish algorithm to extract sets of acoustic features at different temporal resolutions for both datasets.
- Using UMAP, we reduced the acoustic features from the WMD to visualize the dataset structure and explore the relationship between audio recordings and labels describing species taxonomy and geographic locations.
- For the PBD dataset, UMAP visualizations were paired with the use of balanced random forest classifiers fitted on the VGGish acoustic features. With this, we tested how learned acoustic features can be used to identify the biophonic (humpback whale vocalizations) and geophonic (wind speed, surface temperature, and current speed) components of the distal soundscape of Placentia Bay.

**FIGURE 1:**
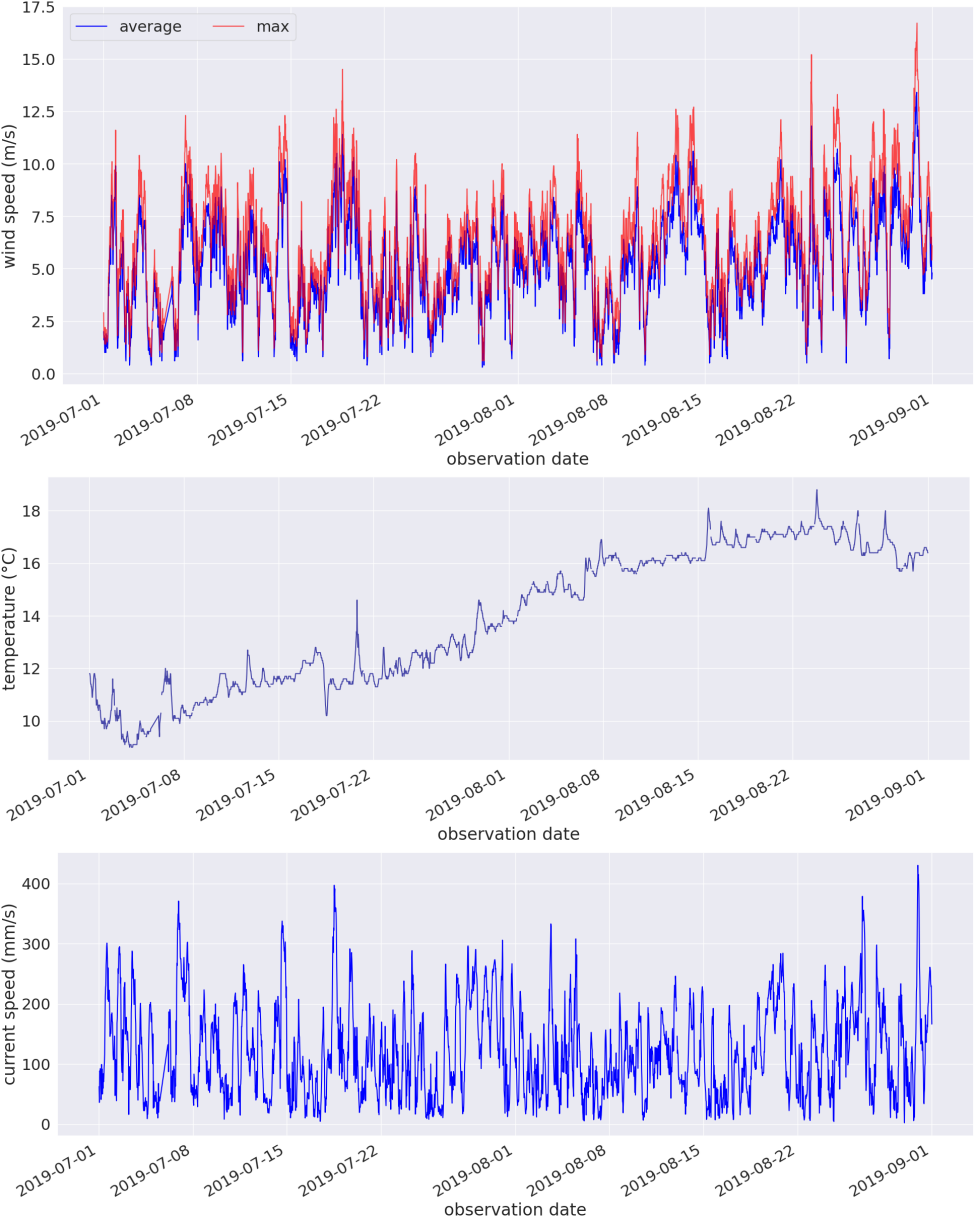
Time series of the environmental variables for the months of July and August 2019. Wind speed (top), ocean surface temperature (middle), and current speed (bottom). All data were obtained from the Red Island SmartAtlantic oceanographic buoy.

This approach is not tied to a specific environment or group of species and can be used to simultaneously investigate the macro‐ and microcharacteristics of marine soundscapes.

## 2 MATERIALS AND METHODS

### 2.1 Data acquisition and preparation

We collected all records available in the Watkins Marine Mammal Database (Watkins Marine Mammal Sound Database, Woods Hole Oceanographic Institution and the New Bedford Whaling Museum) website listed under the “all cuts” page. For each audio file in the WMD the associated metadata included a label for the sound sources present in the recording (biological, anthropogenic, and environmental), as well as information related to the location and date of recording. To minimize the presence of unwanted sounds in the samples, we retained only audio files with a single source listed in the metadata. We then labeled the selected audio clips according to taxonomic group (*Odontocetae*, *Mysticetae*) and species.

We limited the analysis to 12 marine mammal species by discarding data when a species: had less than 60 s of audio available, had a vocal repertoire extending beyond the resolution of the acoustic classification model (VGGish), or was recorded in a single country. To determine if a species was suited for analysis using VGGish, we inspected the Mel‐spectrograms of 3‐s audio samples and retained only species with vocalizations that could be captured in the Mel‐spectrogram (Appendix S1). The vocalizations of species that produce very low frequency or very high frequency were not captured by the Mel‐spectrogram; thus, we removed them from the analysis. To ensure that records included the vocalizations of multiple individuals for each species, we considered only species with records from two or more different countries. Finally, to avoid overrepresentation of sperm whale vocalizations, we excluded 30,000 sperm whale recordings collected in the Dominican Republic. The resulting dataset consisted in 19,682 audio clips with a duration of 960 ms each (0.96 s) (Table 1).

**TABLE 1:** List of species selected from the WMD and corresponding sample sizes (*N* = number of 0.96 s audio samples for each species).

Species	Location (year)	*N*	Total
Bowhead whale	Canada (1988)	705	772
	United States (1972, 1980)	67	
Beluga	Canada (1949, 1962, 1965)	153	224
	United States (1963, 1965, 1968)	71	
Southern right whale	Argentina (1979)	99	109
	Australia (1983)	10	
North Atlantic right whale	Canada (1981)	205	376
	United States (1956, 1959, 1970, 1974)	171	
Short finned pilot whale	Bahamas (1957, 1961)	576	696
	Canada (1958, 1965, 1966, 1967)	83	
	St. Vincents and the Grenadines (1981)	37	
Long finned pilot whale	Canada (1954, 1975)	1154	2029
	Italy (1994)	26	
	North Atlantic Ocean (1975)	166	
	United States (1977)	426	
	Unknown (1975)	257	
Humpback whale	Bahamas (1952, 1955, 1958, 1963)	4819	5601
	Puerto Rico (1954)	6	
	British Virgin Islands (1992)	254	
	United States (1975, 1979, 1980)	269	
	Unknown (1954, 1961)	253	
Orca	Canada (1961, 1964, 1966, 1979)	492	4416
	Norway (1989, 1992)	1696	
	United States (1960, 1997)	2228	
Sperm whale	Bahamas (1952)	4	4368
	Italy (1985, 1988, 1994)	1143	
	Madeira (1966)	1	
	Malta (1985)	220	
	Canada (1975)	966	
	Canary Islands (1987)	7	
	St. Vincents and the Grenadines (1983)	18	
	United States (1972)	1954	
	Unknown (1961, 1962, 1963, 1975)	55	
Rough‐thooted dolphin	Italy (1985)	67	75
	Malta (1985)	8	
Clymene dolphin	Santa Lucia (1983)	286	907
	St. Vincents and the Grenadines (1983)	621	
Bottlenose Dolphin	Croatia (1994)	58	109
	United States (1951, 1984, 1989)	38	
	Unknown (1956)	13	

The Placentia Bay Database (PBD) includes recordings collected by Fisheries and Oceans Canada in Placentia Bay (Newfoundland, Canada), in 2019. The dataset consisted of 2 months of continuous recordings (1230 h), starting on July 1, 2019, and ending on August 31, 2019. The data was collected using an AMAR G4 hydrophone (sensitivity: −165.02 dB re 1 V/μPa at 250 Hz) deployed at 64 m of depth. The hydrophone was set to operate following 15 min cycles, with the first 60 s sampled at 512 kHz, and the remaining 14 min sampled at 64 kHz. For the purpose of this study, we limited the analysis to the 64 kHz recordings.

### 2.2 Acoustic feature extraction

The audio files from the WMD and PBD databases were used as input for VGGish (Abu‐El‐Haija et al., 2016; Chung et al., 2018), a CNN developed and trained to perform general acoustic classification. VGGish was trained on the Youtube8M dataset, containing more than two million user‐labeled audio‐video files. Rather than focusing on the final output of the model (i.e., the assigned labels), here the model was used as a feature extractor (Sethi et al., 2020). VGGish converts audio input into a semantically meaningful vector consisting of 128 features. The model returns features at multiple resolution: ~1 s (960 ms); ~5 s (4800 ms); ~1 min (59,520 ms); ~5 min (299,520 ms). All of the visualizations and results pertaining to the WMD were prepared using the finest feature resolution of ~1 s. The visualizations and results pertaining to the PBD were prepared using the ~5 s features for the humpback whale detection example and were then averaged to an interval of 30 min in order to match the temporal resolution of the environmental measures available for the area.

### 2.3 UMAP ordination and visualization

UMAP is a non‐linear dimensionality reduction algorithm based on the concept of topological data analysis, which, unlike other dimensionality reduction techniques (e.g., tSNE), preserves both the local and global structure of multivariate datasets (McInnes et al., 2018). To allow for data visualization and to reduce the 128 features to two dimensions for further analysis, we applied Uniform Manifold Approximation and Projection (UMAP) to both datasets and inspected the resulting plots.

The UMAP algorithm generates a low‐dimensional representation of a multivariate dataset while maintaining the relationships between points in the global dataset structure (i.e., the 128 features extracted from VGGish). Each point in a UMAP plot in this paper represents an audio sample with duration of ~1 second (WMD dataset), ~ 5 s (PBD dataset, humpback whale detections), or 30 min (PBD dataset, environmental variables). Each point in the two‐dimensional UMAP space also represents a vector of 128 VGGish features. The nearer two points are in the plot space, the nearer the two points are in the 128‐dimensional space, and thus the distance between two points in UMAP reflects the degree of similarity between two audio samples in our datasets. Areas with a high density of samples in UMAP space should, therefore, contain sounds with similar characteristics, and such similarity should decrease with increasing point distance. Previous studies illustrated how VGGish and UMAP can be applied to the analysis of terrestrial acoustic datasets (Heath et al., 2021; Sethi et al., 2020). The visualizations and classification trials presented here illustrate how the two techniques (VGGish and UMAP) can be used together for marine ecoacoustics analysis. UMAP visualizations were prepared using the using the umap‐learn package for python programming language (version 3.10). All UMAP visualizations presented in this study were generated using the algorithm's default parameters.

### 2.4 Labeling sound sources

The labels for the WMD records (i.e., taxonomic group, species, location) were obtained from the database metadata.

For the PBD recordings, we obtained measures of wind speed, surface temperature, and current speed (Figure 1) from an oceanographic buy located in proximity of the recorder.^2^ We choose these three variables for their different contributions to background noise in marine environments. Wind speed contributes to underwater background noise at multiple frequencies, ranging 500 Hz to 20 kHz (Hildebrand et al., 2021). Sea surface temperature contributes to background noise at frequencies between 63 and 125 Hz (Ainslie et al., 2021), while ocean currents contribute to ambient noise at frequencies below 50 Hz (Han et al., 2021) Prior to analysis, we categorized the environmental variables and assigned the categories as labels to the acoustic features (Table 2).

**TABLE 2:** Summary of the BRF models. Variables, labels, number of samples, the n_estimators value selected during cross‐validation, and balanced accuracy scores (Equation 1) are reported for the four BRF models.

Variable	Labels	Number of samples	*n* estimators	Balanced accuracy
Wind speed	0–4 m/s	986	150	0.72
	4–6 m/s	906		
	6–8 m/s	746		
	8–16 m/s	304		
Surface temperature	8–10°C	148	200	0.41
	10–12°C	806		
	12–14°C	478		
	14–16°C	445		
	16–18°C	980		
Current speed	0–20 mm/s	148	200	0.35
	20–60 mm/s	590		
	60–110 mm/s	735		
	110–170 mm/s	733		
	170–260 mm/s	587		
	260–400 mm/s	148		
Humpback whale vocalizations	Absent (0)	3279	200	0.84
	Present (1)	181		

Humpback whale vocalizations in the PBD recordings were processed using the humpback whale acoustic detector created by NOAA and Google (Allen et al., 2021), providing a model score for every ~5 s sample. This model was trained on a large dataset (14 years and 13 locations) using humpback whale recordings annotated by experts (Allen et al., 2021). The model returns scores ranging from 0 to 1, indicating the confidence in the predicted humpback whale presence. We used the results of this detection model to label the PBD samples according to presence of humpback whale vocalizations. To verify the model results, we inspected all audio files that contained a 5 s sample with a model score higher than 0.9 for the month of July. If the presence of a humpback whale was confirmed, we labeled the segment as a model detection. We labeled any additional humpback whale vocalization present in the inspected audio files as a visual detection, while we labeled other sources and background noise samples as absences. In total, we labeled 4.6 h of recordings. We reserved the recordings collected in August to test the precision of the final predictive model.

### 2.5 Label prediction performance

We used Balanced Random Forest models (BRF) provided in the imbalanced‐learn python package (Lemaître et al., 2017) to predict humpback whale presence and environmental conditions from the acoustic features generated by VGGish. We choose BRF as the algorithm as it is suited for datasets characterized by class imbalance. The BRF algorithm performs under sampling of the majority class prior to prediction, allowing to overcome class imbalance (Lemaître et al., 2017). For each model run, the PBD dataset was split into training (80%) and testing (20%) sets.

The training datasets were used to fine‐tune the models through a nested k‐fold cross validation approach with 10‐folds in the outer loop and five‐folds in the inner loop. We selected nested cross validation as it allows optimizing model hyperparameters and performing model evaluation in a single step. We used the default parameters of the BRF algorithm, except for the ‘n_estimators’ hyperparameter, for which we tested five different possible values: 25, 50, 100, 150, and 200. We choose to optimize the model for ‘n_estimators’ as this parameter determines the number of decision trees generated by the BRF model and finding an optimal value reduces the chances of overfitting. Every iteration of the outer loop generates a new train‐validation split of the test dataset, which is then used as input to a BRF.

The testing datasets were then used to evaluate model performance. We evaluated model performance using the balanced‐accuracy score, computed as

(1) $$\text{Balanced Accuracy}\;\left(\mathrm{BA}\right)=\frac{\text{Sensitivity}+\text{Specificity}}{2}$$

We choose balanced‐accuracy scores as the evaluation metric for both datasets as it is suited for measuring model performance when samples are highly imbalanced (Brodersen et al., 2010).

In total, we conducted four trials on the PBD dataset. In the first three trials, we used the PBD dataset to test the ability of VGGish in predicting one of the three environmental variables: wind speed, ocean surface temperature, and current speed. In the fourth trial, we tested the ability of VGGish in identifying humpback whale vocalizations. Finally, we tested the humpback whale model on the recordings from the month of August, which were not part of model training and evaluation. We inspected all detections in August and computed model precision as

(2) $$\text{Precision}=\frac{\text{True Positives}}{\left(\text{True Positives}\;+\;\text{False Negatives}\right)}$$

All predictive models for the PBD were trained and tested on the 128 acoustic features generated by VGGish. The UMAP plots were used to visually inspect the structure of the PBD and WMD features datasets. For the WMD dataset, we used violin plots to explore the distribution of the two UMAP dimensions in relation to the clusters of data points labeled according to taxonomic group, species, and location of origin of the corresponding audio samples.

## 3 RESULTS

### 3.1 Watkins marine mammals sounds database

**FIGURE 2:**
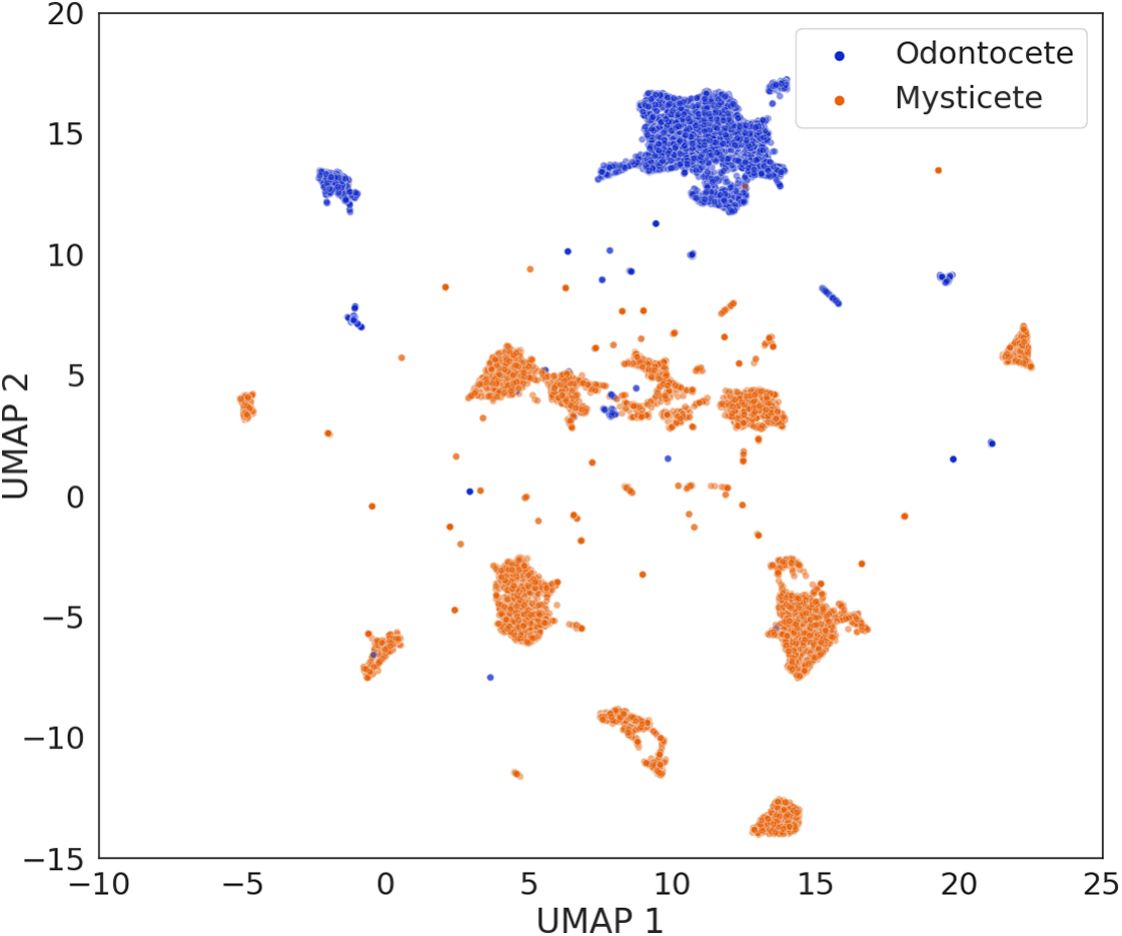
UMAP ordination of the WMD dataset with samples colored according to two marine mammals' taxonomic groups: Mysticete and Odontocete.

The UMAP visualizations of the WMD features showed a complex structure that reflected both taxonomic labels (group and species) and locations. At the macroscale, UMAP separated samples according to the taxonomic group label. Samples belonging to the mysticete and odontocete species occupied two distinct regions of the plot, with little overlap (Figure 2). When looking at the distribution of the two UMAP dimension, this separation was more evident along the second UMAP dimensions, while samples had a higher degree of overlapping values along the first dimension (Appendix S2, Figure S2.1).

**FIGURE 3:**
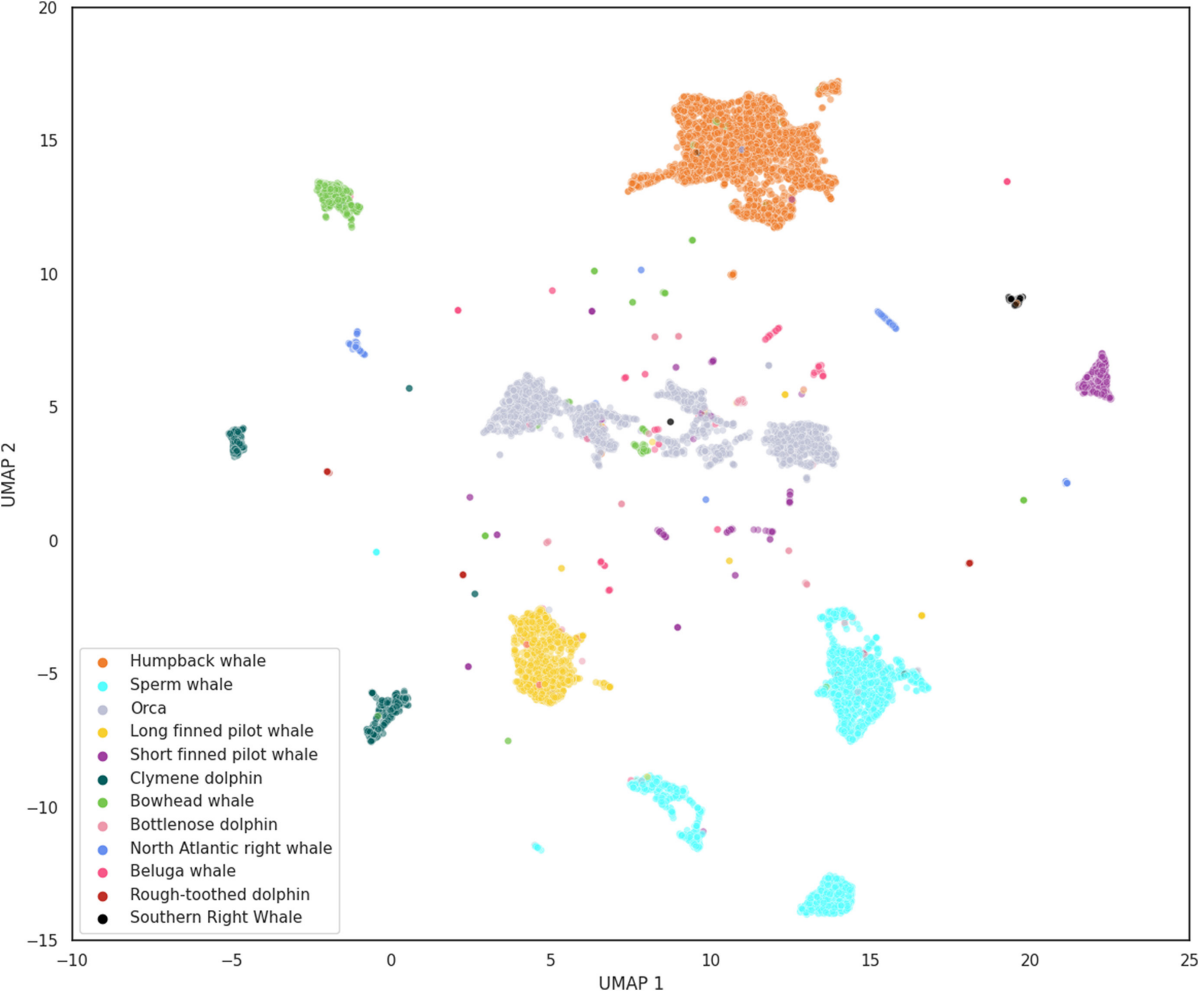
UMAP ordination of the WMD dataset with samples colored according to 12 species of marine mammals.

Of the 12 species considered, eight species formed clear and large clusters: humpback whales, bowhead whales, sperm whales, orcas, long and short finned pilot whales, Clymene dolphins, and North Atlantic right whales (Figure 3). Samples belonging to bottlenose dolphins, beluga whales, rough‐toothed dolphins, and southern right whales, on the other hand, did not form distinct clusters. The distribution of the two UMAP dimensions showed that species were better separated along the second UMAP dimension, while species had overlapping distribution along the first UMAP dimension (Appendix S2, Figure S2.2).

**FIGURE 4:**
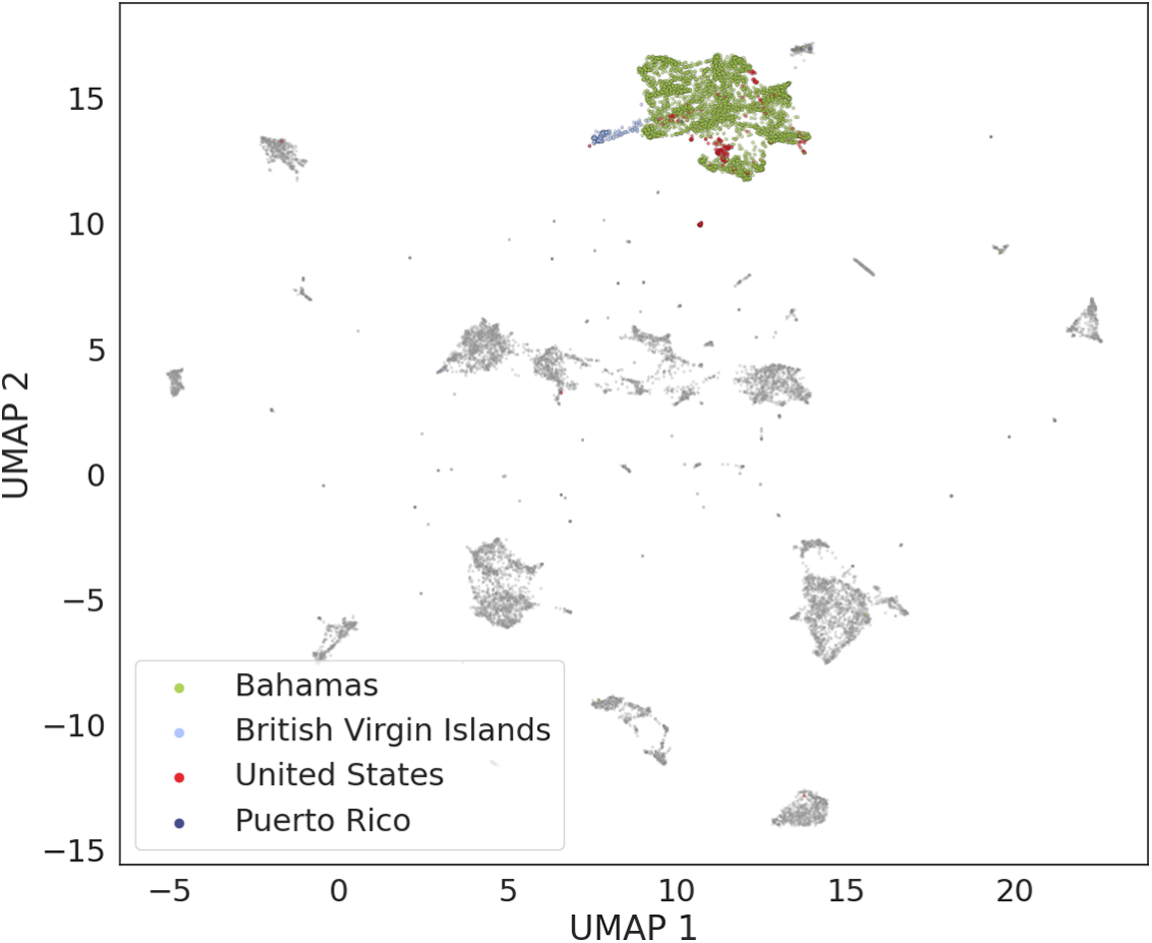
UMAP visualization of the WMD dataset showing humpback whale samples colored according to location.

**FIGURE 5:**
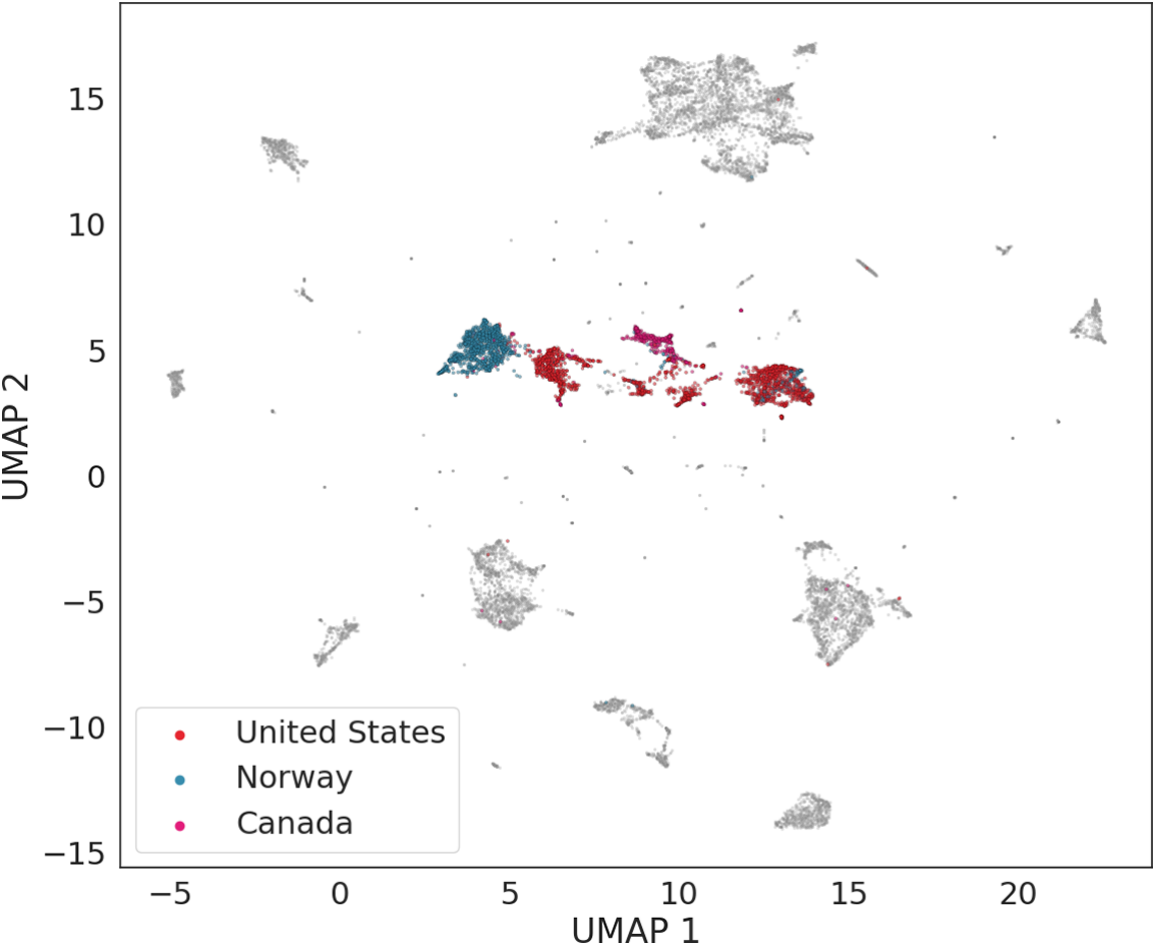
UMAP visualization of the WMD dataset showing orca samples colored according to location.

Samples collected in different locations but belonging to the same species formed close clusters in the UMAP plots. For example, samples of humpback whale vocalizations collected in the Bahamas, the British Virgin Islands, Puerto Rico, and the United States formed a large cluster (Figure 4) with overlapping distributions of the two UMAP dimensions (Appendix S2, Figure S2.3). Similarly, the killer whale samples, collected in the United States, Canada, and Norway, all occupied the same region of the UMAP plot (Figure 5).

### 3.2 Placentia Bay dataset

Results of model parameter selection for the four BRF algorithms fitted on the PBD labels are shown in Table 2.

### 3.3 Oceanographic variables

**FIGURE 6:**
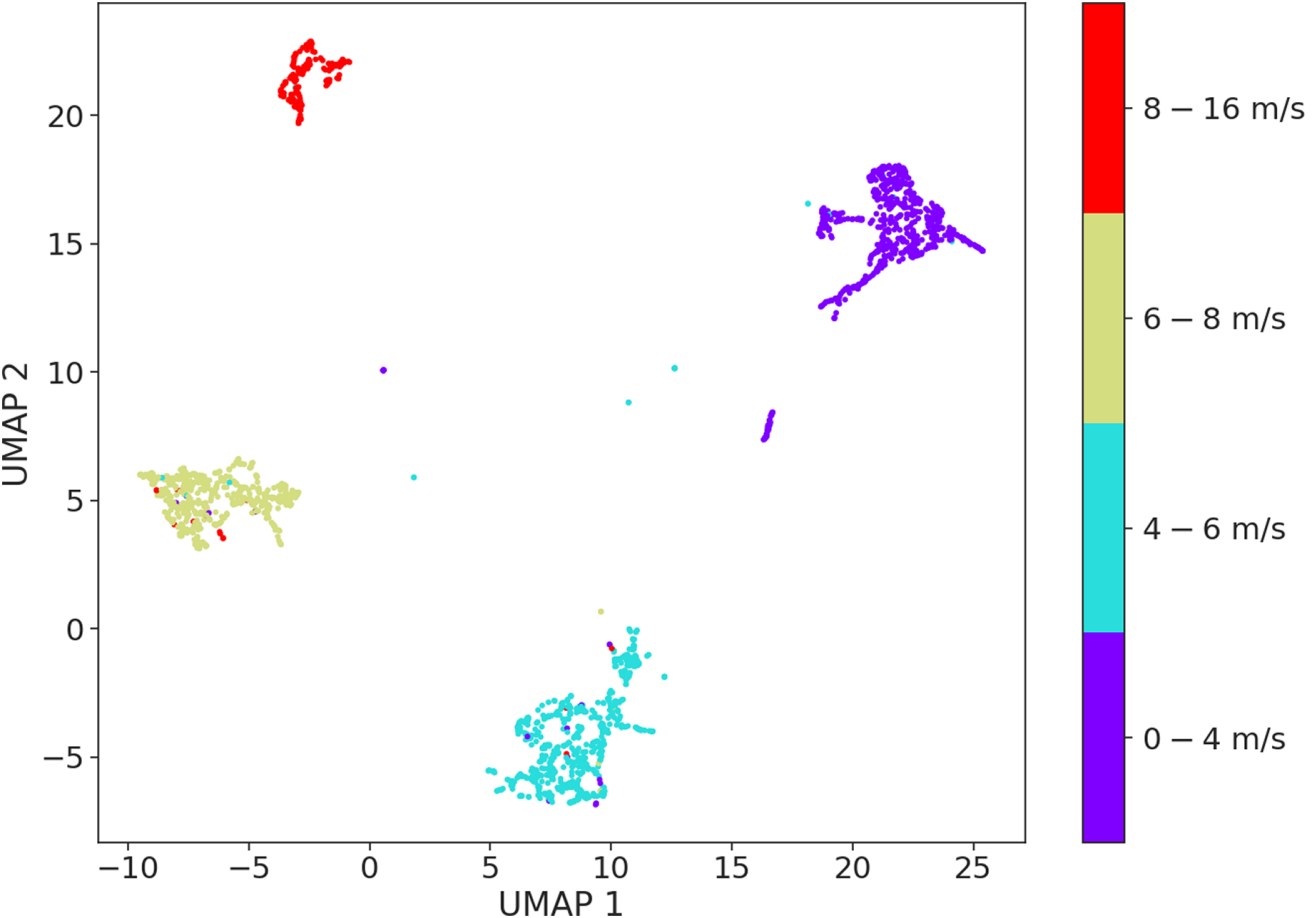
UMAP visualization of the wind speed labels.

Of the three BRF fitted on environmental variables, only the model fitted to the wind speed labels provided overall accurate predictions. This is reflected by the model's balanced accuracy score (0.72) (Table 2). The model accurately discriminated between low (0–4 m/s) and medium (4–6 m/s) wind speeds, while the model ability to correctly classify the higher wind speeds (6–8 m/s and 8–16 m/s) was lower (Appendix S2, Figure S2.4). The BRF models fitted on surface temperature and current speed performed poorly, achieving balanced accuracy scores of 0.41 and 0.35, respectively (Table 2). In the case of temperature, the lowest (8–10°C), the medium (12–14°C), and highest (16–18°C) values were correctly classified for approximately 50% of the testing datasets (Appendix S2, Figure S2.5). In the case of current speed, only the lowest (0–20 mm/s) and highest (260–400 mm/s) were correctly classified for approximately 60% of the dataset (Appendix S2, Figure S2.6). These results are reflected in the UMAP visualizations for the oceanographic variables. Samples labeled by wind speed formed clear and separated clusters (Figure 6). Samples labeled by surface temperature and current speed did not show clear clusters separating the acoustic samples (Appendix S2, Figures S2.8 and S2.9).

### 3.4 Humpback whale detections

**FIGURE 7:**
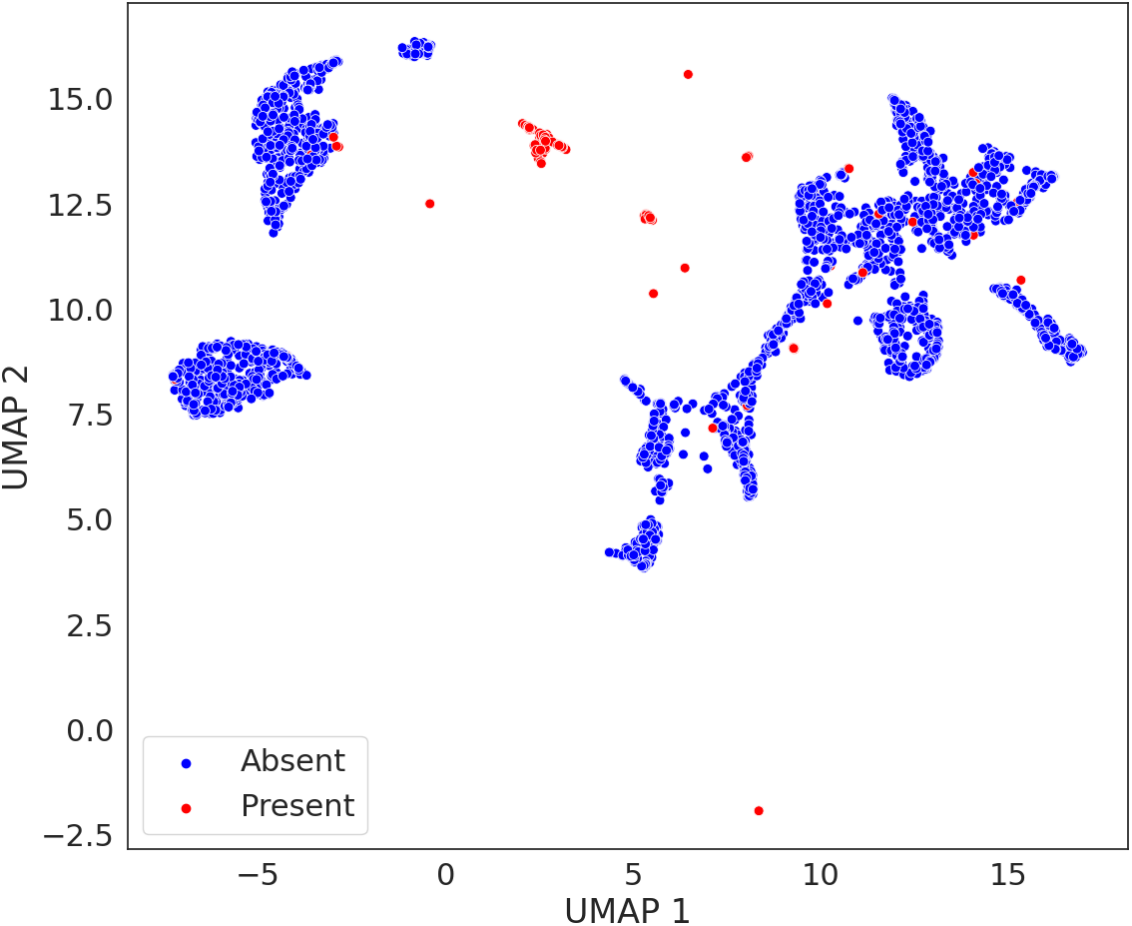
UMAP visualization of the humpback whale labels.

The BRF fitted on the humpback whale labels achieved a balanced accuracy score of 0.84 (Table 2) and showed similar performance for both the presence and absence labels (Appendix S2, Figure S2.7). The UMAP visualization for the humpback whale labels showed a clear cluster of presences (Figure 7). However, several presences plotted within the clusters formed by samples labeled as absences, and a few samples were located between the absences and the presences clusters.

Finaly, the humpback whale BRF model, trained and tested on PBD samples collected in July, predicted 19 presences when run on the samples collected in August. Of these, 15 samples were true presences while the remaining four were false presences, resulting in a precision score of 0.79. All predicted presences were limited to the 23rd of August.

## 4 DISCUSSION

Managing the wellbeing of ecosystems requires identifying when and where human activities are impacting species' occurrence, movement, and behavior. PAM is a useful approach for the detection of both large‐ and small‐scale changes in urban and wild environments, as it allows for continuous and prolonged ecosystem monitoring. Challenges in employing PAM as a standard monitoring tool arise after data collection, when researchers and practitioners need to quickly extract useful information from large acoustic datasets, to understand when and where management actions are needed to preserve the well‐being of ecosystems. The relatively new field of ecoacoustics provides the theoretical background for linking specific characteristics of the acoustic environment to biodiversity and ecosystem health.

The objective of our study was testing how the acoustic features generated by a pre‐trained CNN (VGGish) can be used to link recorded sounds to environmental features and better understand processes happening in marine environments at multiple scales—from changes in oceanographic conditions over the span of months to punctuate events such as the vocalizations produced by marine mammals.

Our analyses revealed several applications for inferring population‐ and location‐specific information from acoustic datasets. The analysis conducted on the WMD dataset shows that the VGGish acoustic features are suited for discriminating between marine mammal species recorded in different environments.

Understanding the evolution of vocal diversity and the role of vocalizations in the ecology of a species is one of the key objectives of bioacoustics research (Luís et al., 2021). Full acoustic repertoires are not available for most species, as building comprehensive lists of vocalizations requires considerable research effort. Here, we show how a general acoustic classification model (VGGish) used as a feature extractor allows us to detect differences and similarities among marine mammal species, without requiring prior knowledge on the species' vocal repertoires. For example, all humpback whale samples formed a compact cluster (Figure 4) and humpback whale populations share common traits in their songs, even when populations are acoustically isolated (Mercado III & Perazio, 2021). Killer whales, on the other hand, formed distinct clusters (Figure 5), and different populations of orcas are characterized by differences in call repertoires and call frequencies (Filatova et al., 2015; Foote & Nystuen, 2008). Although we cannot consider our results as definitive evidence of convergence or divergence in vocal behavior for these two species, we suggest that this aspect should be further investigated, perhaps using more recent recordings of these two species from different populations. Samples from four of the 12 marine mammal species (bottlenose dolphins, beluga whales, rough‐toothed dolphins, and southern right whales) did not form clear clusters. This was most likely due to the low number of samples available for these four species (Table 1).

The analysis conducted on the PBD dataset shows how the VGGish features can be used as a tool to establish relationships between sound recordings and the environment at multiple scales. At the macroscale, the VGGish features were successful in classifying acoustic recordings according to measured wind speeds. This result is particularly useful for determining how winds contribute to underwater background noise. At the fine scale, the VGGish features could be used to identify vocalizations of humpback whales in Placentia Bay. However, presences for the month of August occurred within a single day, indicating that the BRF model may be declaring a large number of samples containing humpback whale vocalizations as absences. Furthermore, the model labeled some of the PBD samples containing only background noise and low‐frequency noise from a passing ship as presences (Appendix S1). The results of the BRF model trained on humpback whale detections could be improved by extending the analysis to longer time frames and to multiple locations, and by including labels for additional sound sources.

Our results highlight a limitation of using a general acoustic classification algorithm trained on recordings collected in terrestrial environments. The audio files used as input in VGGish are limited to a sampling rate of 16 kHz, resulting in a Nyquist frequency of 8 kHz. This is sufficient to capture marine mammal vocalizations that overlap with VGGish frequency limit (Appendix S1), while the method is not suited for species using high‐frequency (e.g., harbor porpoises) or very low‐frequency (e.g., blue and fin whales) vocalizations. This led to the removal of a large number of samples from the WMD dataset. This limitation also explains the poor performance of the models trained on surface temperature and current speed, as their contribution to background noise is evident at frequencies below 125 Hz. Nonetheless, the acoustic features relative to species vocalizing within the 8 kHz range provide useful information relative to the acoustic behavior of marine mammal species. Similarly, the features provided information relative to changes in the acoustic environment of Placentia Bay due to changes in wind speeds. Other CNN approaches, such as AclNet (Huang & Leanos, 2018), allow processing audio with higher sampling rates (e.g., 44.1 kHz) at the cost of increased computing requirements.

Machine‐learned acoustic features respond to multiple marine sound sources and can be employed successfully for investigating both the biological and anthropic components of marine soundscapes (Heath et al., 2021; Sethi et al., 2020). However, their ability to detect species and changes in marine environments is limited by the algorithm's frequency range. A second limitation is that acoustic features are not a plug and play product, as establishing links between features and relevant ecological variables requires additional analyses and data sources. The objective of this study was to explore the application of the methods proposed by Sethi et al. (2020) in a new and unexplored context—the analysis of underwater soundscapes. This approach was particularly suitable for our study as the acoustic samples are not pre‐processed to remove background noises. This approach has also been demonstrated to be resilient to the use of multiple recording devices, as well as to different levels of compression and recording schedules (Heath et al., 2021; Sethi et al., 2020), making it ideal for the analysis of the WMD dataset. An alternative approach where datasets of spectrogram images are directly used as input to dimensionality reduction algorithms is provided by Sainburg et al. (2020) and Thomas et al. (2022). However, this approach relies on removing background noise from the recordings, which, in the case of our study, would have led to loss of information relative to the relationship between environments and acoustic recordings.

By presenting a set of examples focused on marine mammals, we have demonstrated the benefits and challenges of implementing acoustic features as descriptors of marine acoustic environments. Our future research will extend feature extraction and testing to full PAM datasets spanning several years and inclusive of multiple hydrophone deployment locations. Other aspects warranting further investigation are how acoustic features perform when the objective is discriminating vocalizations of individuals belonging to the same species or population, as well as their performance in identifying samples with multiple active sound sources.

Acoustic features are abstract representations of PAM recordings that preserve the original structure and underlying relationships between the original samples, and, at the same time, are a broadly applicable set of metrices that can be used to answer ecoacoustics, ecology, and conservation questions. As such, they can help us understand how natural systems interact with, and respond to, anthropogenic pressures across multiple environments. Finally, the universal nature of acoustic features analysis could help bridge the gap between terrestrial and marine soundscape research. This approach could deepen our understanding of natural systems by enabling multi‐system environmental assessments, allowing researchers to investigate and monitor, for example, how stressor‐induced changes in one system may manifest in another. These benefits accrue from an approach that is more objective than manual analyses and requires far less human effort.

## AUTHOR CONTRIBUTIONS

**Simone Cominelli:** Conceptualization (lead); data curation (equal); formal analysis (equal); investigation (equal); methodology (equal); software (lead); validation (lead); visualization (lead); writing – original draft (lead); writing – review and editing (lead). **Nicolo' Bellin:** Conceptualization (lead); data curation (lead); formal analysis (lead); investigation (lead); methodology (lead); software (lead); validation (lead); visualization (lead); writing – original draft (lead); writing – review and editing (lead). **Carissa D. Brown:** Funding acquisition (lead); project administration (lead); supervision (lead); writing – review and editing (equal). **Valeria Rossi:** Funding acquisition (supporting); project administration (supporting); supervision (lead); writing – review and editing (supporting). **Jack Lawson:** Funding acquisition (lead); project administration (lead); resources (lead); supervision (lead); writing – review and editing (supporting).

## CONFLICT OF INTEREST STATEMENT

The authors declare that there are no conflicts of interest.

## Supporting information


Appendix S1.

Appendix S2.

## Data Availability

Scripts to reproduce the images and analysis results reported here, and tables containing the VGGish acoustic features and labels for the two datasets can be found at the following links:
Dryad (data tables): https://doi.org/10.5061/dryad.3bk3j9kn8
Zenodo (python scripts): https://doi.org/10.5281/zenodo.10019845

## ENDNOTES

^1^

https://cis.whoi.edu/science/B/whalesounds/index.cfm.

^2^

https://www.smartatlantic.ca/station_alt.html?id=placentiabay_redisland.

## References

[ece310951-bib-0001] Abu‐El‐Haija, S. , Kothari, N. , Lee, J. , Natsev, P. , Toderici, G. , Varadarajan, B. , & Vijayanarasimhan, S. (2016). YouTube‐8M: A large‐scale video classification benchmark. *arXiv*. https://arxiv.org/abs/1609.08675

[ece310951-bib-0002] Ainslie, M. A. , Andrew, R. K. , Howe, B. M. , & Mercer, J. A. (2021). Temperature‐driven seasonal and longer term changes in spatially averaged deep ocean ambient sound at frequencies 63–125 Hz. The Journal of the Acoustical Society of America, 149(4), 2531–2545. 10.1121/10.0003960 33940862

[ece310951-bib-0003] Ainsworth, T. D. , Hurd, C. L. , Gates, R. D. , & Boyd, P. W. (2020). How do we overcome abrupt degradation of marine ecosystems and meet the challenge of heat waves and climate extremes? Global Change Biology, 26(2), 343–354. 10.1111/gcb.14901 31873988

[ece310951-bib-0004] Allen, A. N. , Harvey, M. , Harrell, L. , Jansen, A. , Merkens, K. P. , Wall, C. C. , Cattiau, J. , & Oleson, E. M. (2021). A convolutional neural network for automated detection of humpback whale song in a diverse, long‐term passive acoustic dataset. Frontiers in Marine Science, 8, 1–12. 10.3389/fmars.2021.607321 35685121

[ece310951-bib-0005] Almeira, J. , & Guecha, S. (2019). Dominant power spectrums as a tool to establish an ecoacoustic baseline in a premontane moist forest. Landscape and Ecological Engineering, 15(1), 121–130. 10.1007/s11355-018-0355-0

[ece310951-bib-0006] Bergler, C. , Schröter, H. , Cheng, R. X. , Barth, V. , Weber, M. , Nöth, E. , Hofer, H. , & Maier, A. (2019). ORCA‐SPOT: An automatic killer whale sound detection toolkit using deep learning. Scientific Reports, 9(1), 10997. 10.1038/s41598-019-47335-w 31358873 PMC6662697

[ece310951-bib-0007] Bittle, M. , & Duncan, A. (2013). A review of current marine mammal detection and classification algorithms for use in automated passive acoustic monitoring. *Proceedings of Acoustics*, November, 1–8.

[ece310951-bib-0008] Brodersen, K. H. , Ong, C. S. , Stephan, K. E. , & Buhmann, J. M. (2010). The balanced accuracy and its posterior distribution. In *2010 20th international conference on pattern recognition* (pp. 3121–3124). 10.1109/ICPR.2010.764

[ece310951-bib-0009] Christin, S. , Hervet, É. , & Lecomte, N. (2019). Applications for deep learning in ecology. Methods in Ecology and Evolution, 10(10), 1632–1644. 10.1111/2041-210X.13256

[ece310951-bib-0010] Chung, C. , Patel, S. , Lee, R. , Fu, L. , Reilly, S. , Ho, T. , Lionetti, J. , George, M. D. , & Taylor, P. (2018). Implementation of an integrated computerized prescriber order‐entry system for chemotherapy in a multisite safety‐net health system. American Journal of Health‐System Pharmacy, 75(6), 398–406. 10.2146/ajhp170251 29523537

[ece310951-bib-0011] Clink, D. J. , & Klinck, H. (2021). Unsupervised acoustic classification of individual gibbon females and the implications for passive acoustic monitoring. Methods in Ecology and Evolution, 12(2), 328–341. 10.1111/2041-210X.13520

[ece310951-bib-0012] Dong, X. , Yan, N. , & Wei, Y. (2018). Insect sound recognition based on convolutional neural network. In *2018 IEEE 3rd international conference on image, vision and computing (ICIVC)* (pp. 855–859). 10.1109/ICIVC.2018.8492871

[ece310951-bib-0013] Durette‐Morin, D. , Davies, K. T. A. , Johnson, H. D. , Brown, M. W. , Moors‐Murphy, H. , Martin, B. , & Taggart, C. T. (2019). Passive acoustic monitoring predicts daily variation in North Atlantic right whale presence and relative abundance in Roseway Basin, Canada. Marine Mammal Science, 35(4), 1280–1303. 10.1111/mms.12602

[ece310951-bib-0014] Eftestøl, S. , Flydal, K. , Tsegaye, D. , & Colman, J. E. (2019). Mining activity disturbs habitat use of reindeer in Finnmark, Northern Norway. Polar Biology, 42(10), 1849–1858. 10.1007/s00300-019-02563-8

[ece310951-bib-0015] Elise, S. , Bailly, A. , Urbina‐Barreto, I. , Mou‐Tham, G. , Chiroleu, F. , Vigliola, L. , Robbins, W. D. , & Bruggemann, J. H. (2019). An optimised passive acoustic sampling scheme to discriminate among coral reefs' ecological states. Ecological Indicators, 107, 105627. 10.1016/j.ecolind.2019.105627

[ece310951-bib-0016] Erbe, C. , Marley, S. A. , Schoeman, R. P. , Smith, J. N. , Trigg, L. E. , & Embling, C. B. (2019). The effects of ship noise on marine mammals—A review. Frontiers in Marine Science, 6, 1–21. 10.3389/fmars.2019.00606 36817748

[ece310951-bib-0017] Farina, A. , & Gage, S. H. (2017). Ecoacoustics: The ecological role of sounds. In A. Farina & S. H. Gage (Eds.), Ecoacoustics: The ecological role of sounds. John Wiley & Sons, Ltd.

[ece310951-bib-0018] Filatova, O. A. , Miller, P. J. O. , Yurk, H. , Samarra, F. I. P. , Hoyt, E. , Ford, J. K. B. , Matkin, C. O. , & Barrett‐Lennard, L. G. (2015). Killer whale call frequency is similar across the oceans, but varies across sympatric ecotypes. The Journal of the Acoustical Society of America, 138(1), 251–257. 10.1121/1.4922704 26233024

[ece310951-bib-0019] Fleming, A. H. , Yack, T. , Redfern, J. V. , Becker, E. A. , Moore, T. J. , & Barlow, J. (2018). Combining acoustic and visual detections in habitat models of Dall's porpoise. Ecological Modelling, 384, 198–208. 10.1016/j.ecolmodel.2018.06.014

[ece310951-bib-0020] Foote, A. D. , & Nystuen, J. A. (2008). Variation in call pitch among killer whale ecotypes. Journal of the Acoustical Society of America, 123, 1747–1752. 10.1121/1.2836752 18345862

[ece310951-bib-0021] Geiger, J. T. , Schuller, B. , & Rigoll, G. (2013). Large‐scale audio feature extraction and SVM for acoustic scene classification. 2013 IEEE Workshop on Applications of Signal Processing to Audio and Acoustics, New Paltz, NY, USA, 2013, 1–4. 10.1109/WASPAA.2013.6701857

[ece310951-bib-0022] Gibb, R. , Browning, E. , Glover‐Kapfer, P. , & Jones, K. E. (2019). Emerging opportunities and challenges for passive acoustics in ecological assessment and monitoring. Methods in Ecology and Evolution, 10(2), 169–185. 10.1111/2041-210X.13101

[ece310951-bib-0023] Gómez, W. E. , Isaza, C. V. , & Daza, J. M. (2018). Identifying disturbed habitats: A new method from acoustic indices. Ecological Informatics, 45, 16–25. 10.1016/j.ecoinf.2018.03.001

[ece310951-bib-0024] Grinfeder, E. , Lorenzi, C. , Haupert, S. , & Sueur, J. (2022). What do we mean by “soundscape”? A functional description. Frontiers in Ecology and Evolution, 10, 1–14. 10.3389/fevo.2022.894232

[ece310951-bib-0025] Han, D. G. , Joo, J. , Son, W. , Cho, K. H. , Choi, J. W. , Yang, E. J. , Kim, J. H. , Kang, S. H. , & La, H. S. (2021). Effects of geophony and anthrophony on the underwater acoustic environment in the east Siberian Sea, Arctic Ocean. Geophysical Research Letters, 48(12), 1–9. 10.1029/2021GL093097

[ece310951-bib-0026] Heath, B. E. , Sethi, S. S. , Orme, C. D. L. , Ewers, R. M. , & Picinali, L. (2021). How index selection, compression, and recording schedule impact the description of ecological soundscapes. Ecology and Evolution, 11(19), 13206–13217. 10.1002/ece3.8042 34646463 PMC8495811

[ece310951-bib-0027] Hershey, S. , Chaudhuri, S. , Ellis, D. P. W. , Gemmeke, J. F. , Jansen, A. , Moore, R. C. , Plakal, M. , Platt, D. , Saurous, R. A. , Seybold, B. , Slaney, M. , Weiss, R. J. , & Wilson, K. (2017). CNN architectures for large‐scale audio classification. In 2017 IEEE international conference on acoustics, speech and signal processing (ICASSP), New Orleans, LA, USA, pp. 131–135. 10.1109/ICASSP.2017.7952132

[ece310951-bib-0028] Hildebrand, J. A. , Frasier, K. E. , Baumann‐Pickering, S. , & Wiggins, S. M. (2021). An empirical model for wind‐generated ocean noise. The Journal of the Acoustical Society of America, 149(6), 4516–4533. 10.1121/10.0005430 34241440

[ece310951-bib-0029] Houegnigan, L. , Safari, P. , Nadeu, C. , Van Der Schaar, M. , & Andre, M. (2017). A Novel approach to real‐time range estimation of underwater acoustic sources using supervised machine learning. *OCEANS 2017 – Aberdeen*, *2017*‐*Octob*, 1–5. 10.1109/OCEANSE.2017.8084914

[ece310951-bib-0030] Huang, J. J. , & Leanos, J. J. A. (2018). AclNet: Efficient end‐to‐end audio classification CNN. *arXiv*. http://arxiv.org/abs/1811.06669

[ece310951-bib-0031] Kowarski, K. A. , & Moors‐Murphy, H. (2021). A review of big data analysis methods for baleen whale passive acoustic monitoring. Marine Mammal Science, 37(2), 652–673. 10.1111/mms.12758

[ece310951-bib-0032] Kunc, H. P. , McLaughlin, K. E. , & Schmidt, R. (2016). Aquatic noise pollution: Implications for individuals, populations, and ecosystems. Proceedings of the Royal Society B: Biological Sciences, 283(1836), 20160839. 10.1098/rspb.2016.0839 PMC5013761 27534952

[ece310951-bib-0033] Kunc, H. P. , & Schmidt, R. (2019). The effects of anthropogenic noise on animals: A meta‐analysis. Biology Letters, 15(11), 20190649. 10.1098/rsbl.2019.0649 31744413 PMC6892517

[ece310951-bib-0034] LeBien, J. , Zhong, M. , Campos‐Cerqueira, M. , Velev, J. P. , Dodhia, R. , Ferres, J. L. , & Aide, T. M. (2020). A pipeline for identification of bird and frog species in tropical soundscape recordings using a convolutional neural network. Ecological Informatics, 59, 101113. 10.1016/j.ecoinf.2020.101113

[ece310951-bib-0035] Lemaître, G. , Nogueira, F. , & Aridas, C. K. (2017). Imbalanced‐learn: A python toolbox to tackle the curse of imbalanced datasets in machine learning. Journal of Machine Learning Research, 18(1), 559–563.

[ece310951-bib-0036] Luís, A. R. , May‐Collado, L. J. , Rako‐Gospić, N. , Gridley, T. , Papale, E. , Azevedo, A. , Silva, M. A. , Buscaino, G. , Herzing, D. , & dos Santos, M. E. (2021). Vocal universals and geographic variations in the acoustic repertoire of the common bottlenose dolphin. Scientific Reports, 11(1), 1–9. 10.1038/s41598-021-90710-9 34088923 PMC8178411

[ece310951-bib-0037] Lumini, A. , Nanni, L. , & Maguolo, G. (2019). Deep learning for plankton and coral classification. Applied Computing and Informatics, 19, 265–283. 10.1016/j.aci.2019.11.004

[ece310951-bib-0038] Luo, J. , Siemers, B. M. , & Koselj, K. (2015). How anthropogenic noise affects foraging. Global Change Biology, 21(9), 3278–3289. 10.1111/gcb.12997 26046451

[ece310951-bib-0039] Madhusudhana, S. K. , Chakraborty, B. , & Latha, G. (2019). Humpback whale singing activity off the Goan coast in the eastern Arabian Sea. Bioacoustics, 28(4), 329–344. 10.1080/09524622.2018.1458248

[ece310951-bib-0040] McInnes, L. , Healy, J. , & Melville, J. (2018). UMAP: Uniform Manifold Approximation and Projection for Dimension Reduction. *arXiv*. http://arxiv.org/abs/1802.03426

[ece310951-bib-0041] Mercado, E., III , & Perazio, C. E. (2021). Similarities in composition and transformations of songs by humpback whales (Megaptera novaeangliae) over time and space. Journal of Comparative Psychology, 135(1), 28–50. 10.1037/com0000268.supp 33555905

[ece310951-bib-0042] Mishachandar, B. , & Vairamuthu, S. (2021). Diverse ocean noise classification using deep learning. Applied Acoustics, 181, 108141. 10.1016/j.apacoust.2021.108141

[ece310951-bib-0043] Nguyen Hong Duc, P. , Cazau, D. , White, P. R. , Gérard, O. , Detcheverry, J. , Urtizberea, F. , & Adam, O. (2021). Use of Ecoacoustics to characterize the marine acoustic environment off the North Atlantic French Saint‐Pierre‐et‐Miquelon archipelago. Journal of Marine Science and Engineering, 9(2), 177. 10.3390/jmse9020177

[ece310951-bib-0044] Norouzzadeh, M. S. , Nguyen, A. , Kosmala, M. , Swanson, A. , Palmer, M. S. , Packer, C. , & Clune, J. (2018). Automatically identifying, counting, and describing wild animals in camera‐trap images with deep learning. Proceedings of the National Academy of Sciences of the United States of America, 115(25), E5716–E5725. 10.1073/pnas.1719367115 29871948 PMC6016780

[ece310951-bib-0045] Plourde, S. , Lehoux, C. , Johnson, C. L. , Perrin, G. , & Lesage, V. (2019). North Atlantic right whale (Eubalaena glacialis) and its food: (I) a spatial climatology of Calanus biomass and potential foraging habitats in Canadian waters. Journal of Plankton Research, 41(5), 667–685. 10.1093/plankt/fbz024

[ece310951-bib-0046] Roch, M. A. , Lindeneau, S. , Aurora, G. S. , Frasier, K. E. , Hildebrand, J. A. , Glotin, H. , & Baumann‐Pickering, S. (2021). Using context to train time‐domain echolocation click detectors. The Journal of the Acoustical Society of America, 149(5), 3301–3310. 10.1121/10.0004992 34241092

[ece310951-bib-0047] Root‐Gutteridge, H. , Cusano, D. A. , Shiu, Y. , Nowacek, D. P. , Van Parijs, S. M. , & Parks, S. E. (2018). A lifetime of changing calls: North Atlantic right whales, Eubalaena glacialis, refine call production as they age. Animal Behaviour, 137, 21–34. 10.1016/j.anbehav.2017.12.016

[ece310951-bib-0048] Rycyk, A. M. , Tyson Moore, R. B. , Wells, R. S. , McHugh, K. A. , Berens McCabe, E. J. , & Mann, D. A. (2020). Passive acoustic listening stations (PALS) show rapid onset of ecological effects of harmful algal blooms in real time. Scientific Reports, 10(1), 17863. 10.1038/s41598-020-74647-z 33082430 PMC7575606

[ece310951-bib-0049] Sainburg, T. , Thielk, M. , & Gentner, T. Q. (2020). Finding, visualizing, and quantifying latent structure across diverse animal vocal repertoires. PLoS Computational Biology, 16(10), e1008228. 10.1371/journal.pcbi.1008228 33057332 PMC7591061

[ece310951-bib-0050] Schmidt, R. , Morrison, A. , & Kunc, H. P. (2014). Sexy voices – no choices: Male song in noise fails to attract females. Animal Behaviour, 94, 55–59. 10.1016/j.anbehav.2014.05.018

[ece310951-bib-0051] Sethi, S. S. , Jones, N. S. , Fulcher, B. D. , Picinali, L. , Clink, D. J. , Klinck, H. , Orme, C. D. L. , Wrege, P. H. , & Ewers, R. M. (2020). Characterizing soundscapes across diverse ecosystems using a universal acoustic feature set. Proceedings of the National Academy of Sciences of the United States of America, 117(29), 17049–17055. 10.1073/pnas.2004702117 32636258 PMC7382238

[ece310951-bib-0052] Stowell, D. (2022). Computational bioacoustics with deep learning: A review and roadmap. PeerJ, 10, e13152. 10.7717/peerj.13152 35341043 PMC8944344

[ece310951-bib-0053] Sully, S. , Burkepile, D. E. , Donovan, M. K. , Hodgson, G. , & van Woesik, R. (2019). A global analysis of coral bleaching over the past two decades. Nature Communications, 10(1), 1264. 10.1038/s41467-019-09238-2 PMC6427037 30894534

[ece310951-bib-0054] Tabak, M. A. , Norouzzadeh, M. S. , Wolfson, D. W. , Sweeney, S. J. , Vercauteren, K. C. , Snow, N. P. , Halseth, J. M. , Di Salvo, P. A. , Lewis, J. S. , White, M. D. , Teton, B. , Beasley, J. C. , Schlichting, P. E. , Boughton, R. K. , Wight, B. , Newkirk, E. S. , Ivan, J. S. , Odell, E. A. , Brook, R. K. , … Miller, R. S. (2019). Machine learning to classify animal species in camera trap images: Applications in ecology. Methods in Ecology and Evolution, 10(4), 585–590. 10.1111/2041-210X.13120

[ece310951-bib-0055] Thomas, M. , Jensen, F. H. , Averly, B. , Demartsev, V. , Manser, M. B. , Sainburg, T. , Roch, M. A. , & Strandburg‐Peshkin, A. (2022). A practical guide for generating unsupervised, spectrogram‐based latent space representations of animal vocalizations. Journal of Animal Ecology, 91(8), 1567–1581. 10.1111/1365-2656.13754 35657634

[ece310951-bib-0056] Usman, A. M. , Ogundile, O. O. , & Versfeld, D. J. J. (2020). Review of automatic detection and classification techniques for cetacean vocalization. IEEE Access, 8, 105181–105206. 10.1109/ACCESS.2020.3000477

[ece310951-bib-0057] Vester, H. , Hallerberg, S. , Timme, M. , & Hammerschmidt, K. (2017). Vocal repertoire of long‐finned pilot whales (*Globicephala melas*) in northern Norway. The Journal of the Acoustical Society of America, 141(6), 4289–4299. 10.1121/1.4983685 28618811

[ece310951-bib-0058] Vickers, W. , Milner, B. , Gorpincenko, A. , & Lee, R. (2021). Methods to improve the robustness of right whale detection using CNNs in changing conditions. *2020 28th European signal processing conference (EUSIPCO)*, *2021*‐*January*, 106–110. 10.23919/Eusipco47968.2020.9287565

