# Supplementary material for: Acoustic features as a tool to visualize and explore marine soundscapes: Applications illustrated using marine mammal passive acoustic monitoring datasets
Source: Ecol Evol. 2024 Feb 21;14(2):e10951. doi: 10.1002/ece3.10951 (PMC10880131; doi:10.1002/ece3.10951)
Supplement: Supplementary file 1 — Appendix S1. [file ECE3-14-e10951-s001.docx]

**APPENDIX S1: Spectrograms & Log-Mel Spectrograms by Species**

**S1.1: Spectrograms & Log-Mel Spectrograms by Species – WMD dataset**

Spectrograms and Mel-spectrograms of audio samples form the WMD dataset for 12 marine mammal species . For each example, the sample ID for the audio file corresponds to the record ID from the WMD.

All spectrograms were computed with nftt = 2048 and hop length = 512.

**Sample 1: Humpback whale**

WMD Sample ID: 5801801P

**
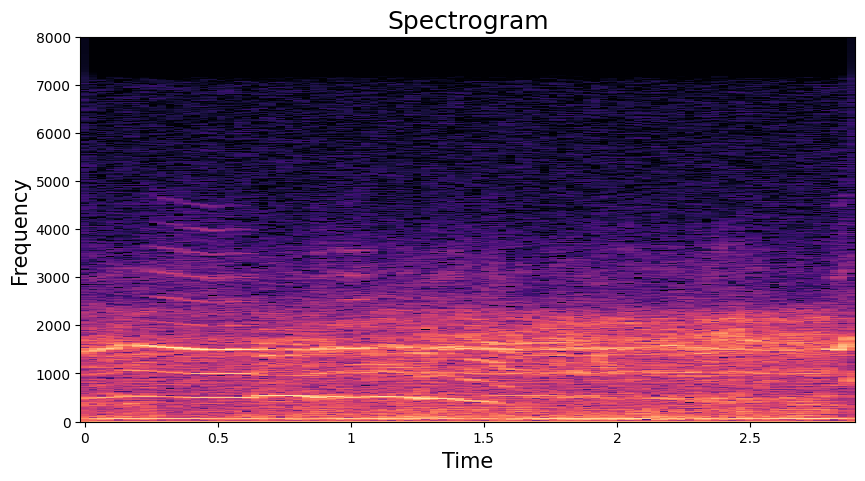

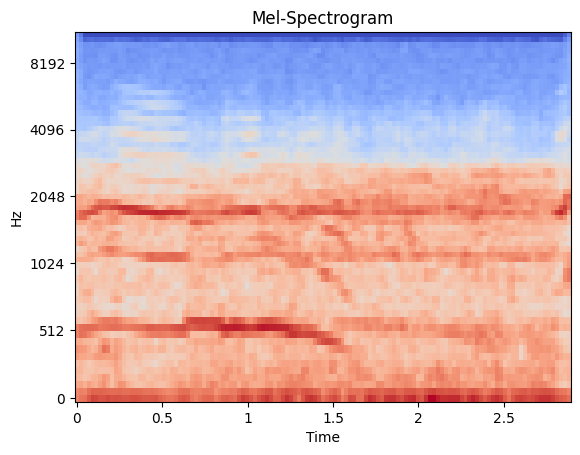
**

**Sample 2 Killer Whale**

WMD Sample ID: 6002602S


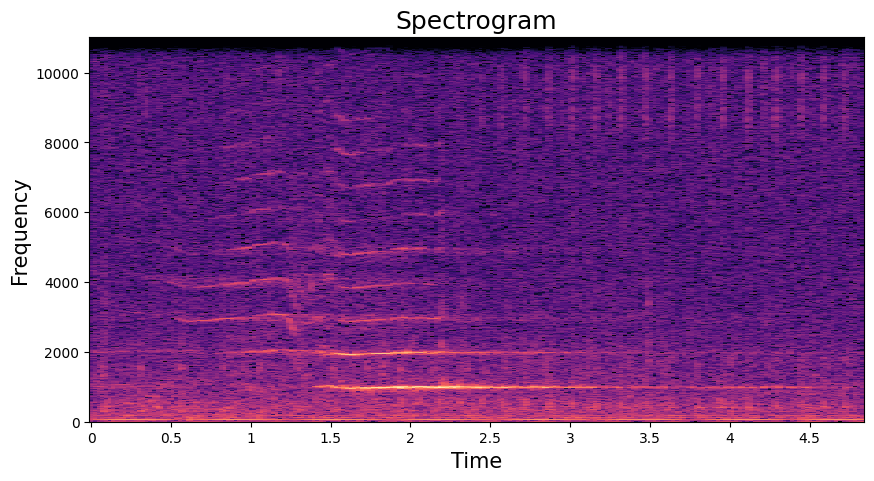


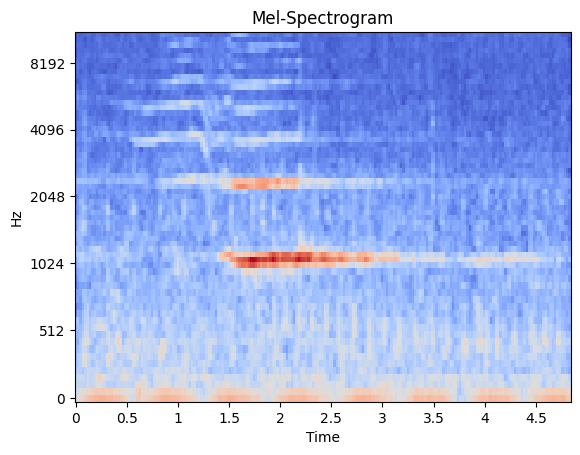


**Sample 3: Bowhead Whale**

WMD Sample ID: 80001004


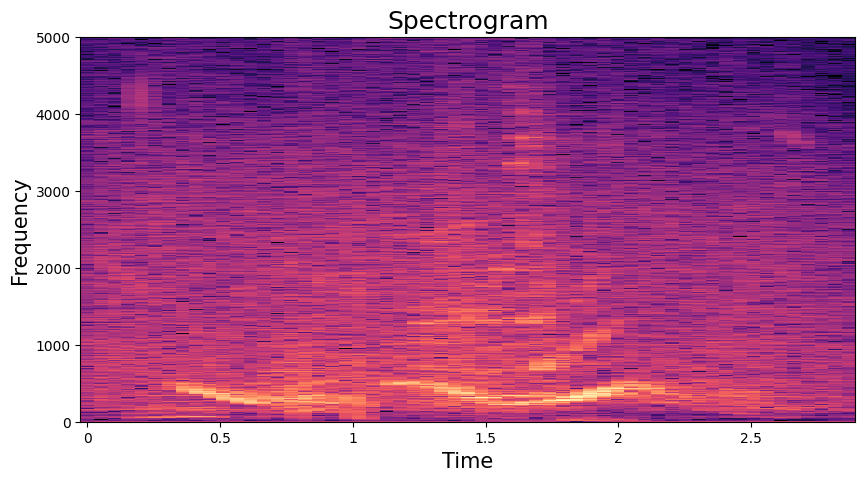


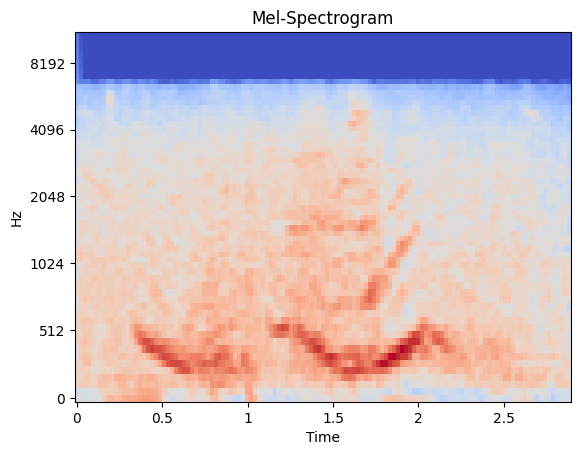


**Sample 4: North Atlantic Right Whale**

WMD Sample ID: 8101301D


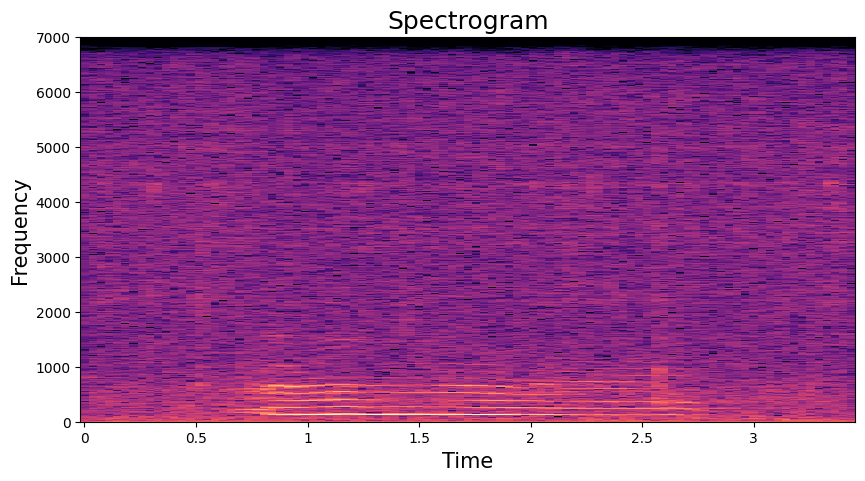


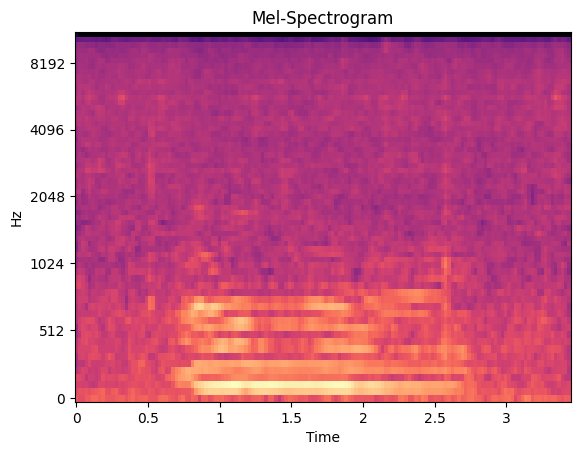


**Sample 5: Southern Right Whale**

WMD Sample ID: 7900200M


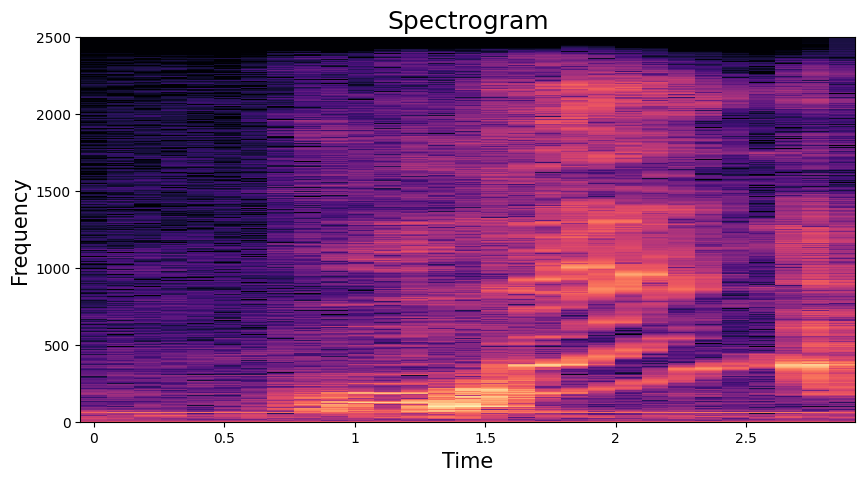

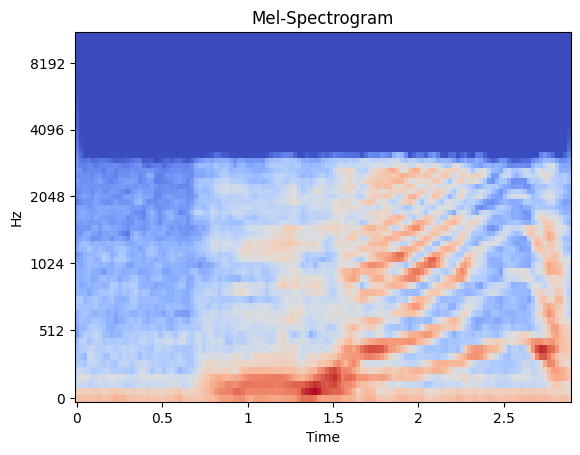


**Sample 6: Long Finned Pilot Whale**

WMD Sample ID: 54024003


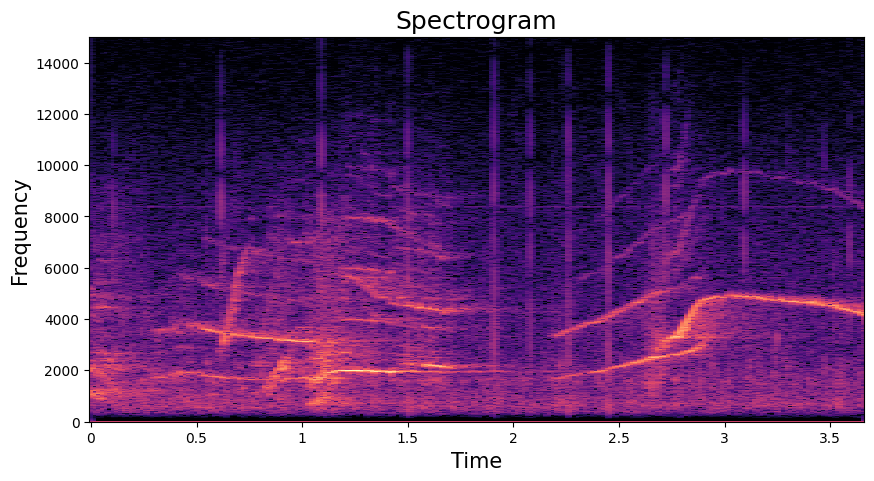


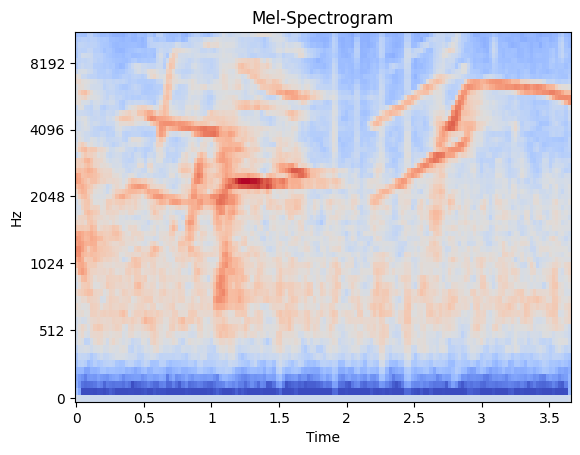


**Sample 7: Short finned pilot whale**

WMD Sample ID: 57021004


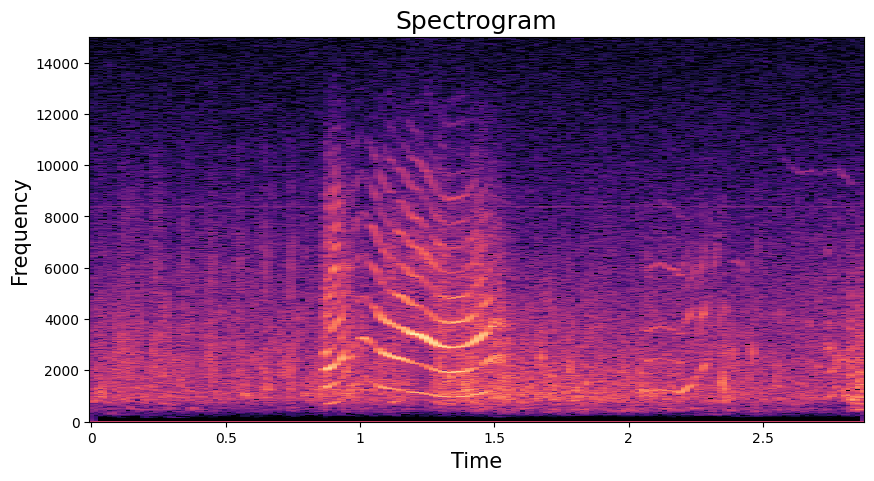


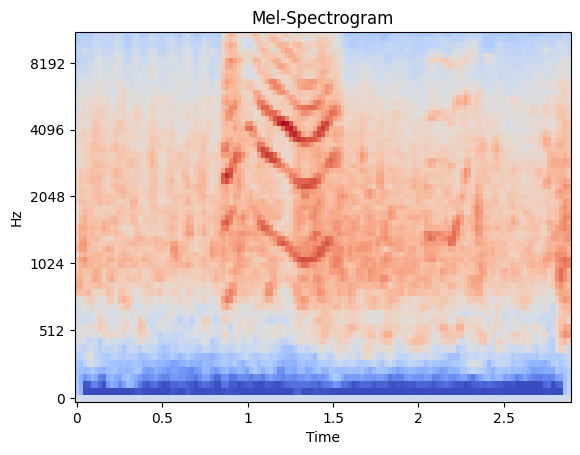


**Sample 8: Rough Toothed Dolphin**

WMD Sample ID: 8501301K


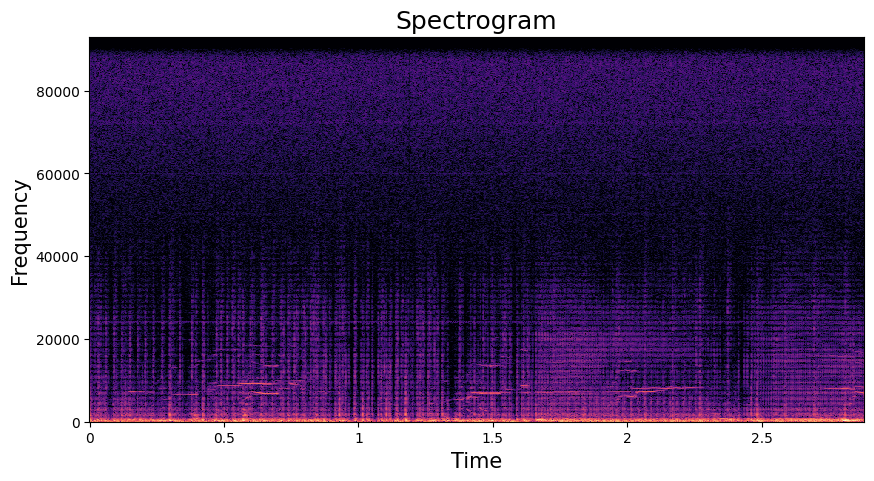


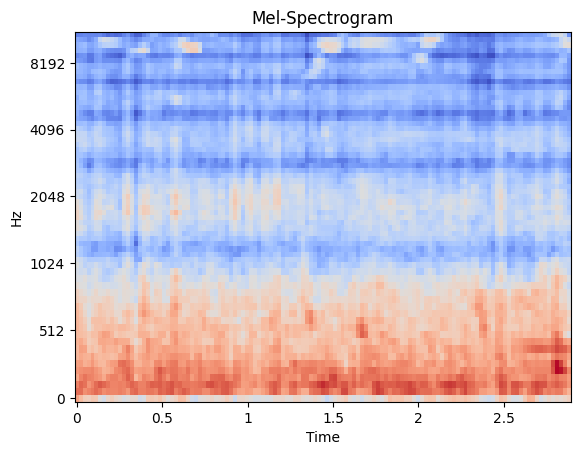


**Sample 9: Clymene Dolphin**

WMD Sample ID: 8300601S


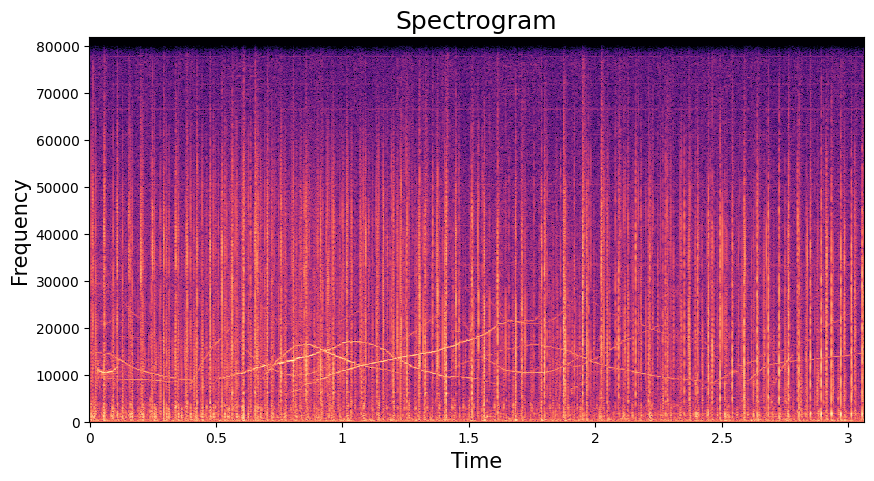

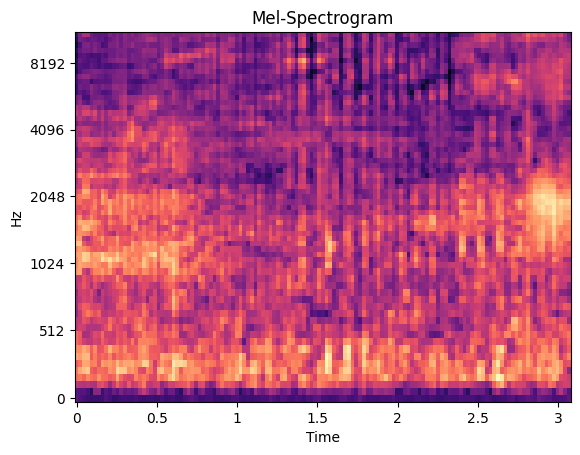


**Sample 10: Beluga whale**

WMD Sample ID: 62019004


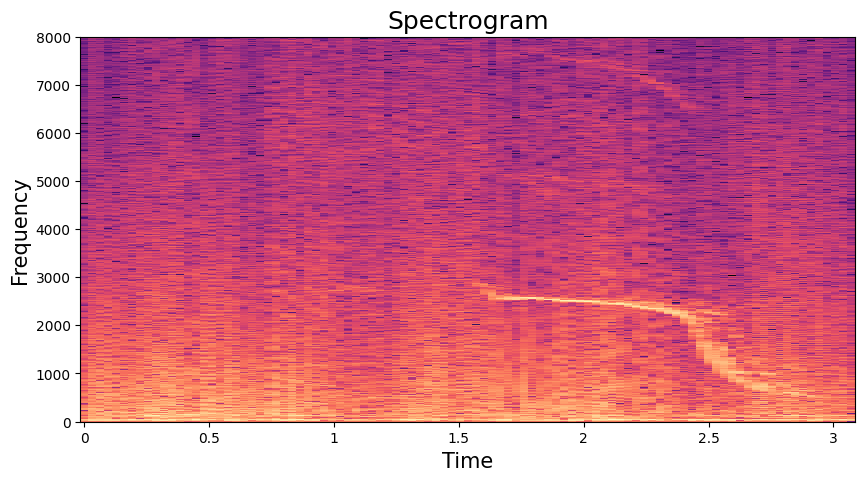


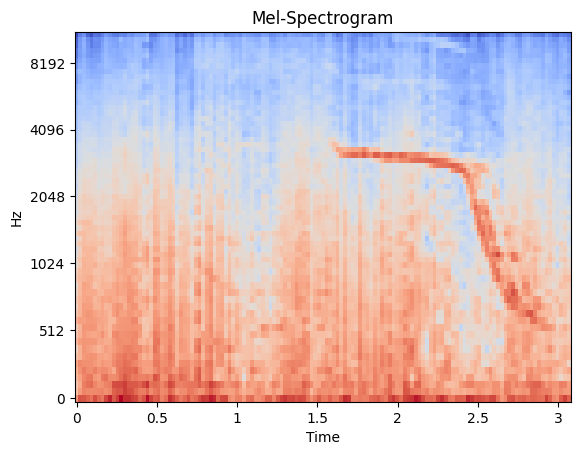


**Sample 11: Sperm whale**

WMD Sample ID: 72009001


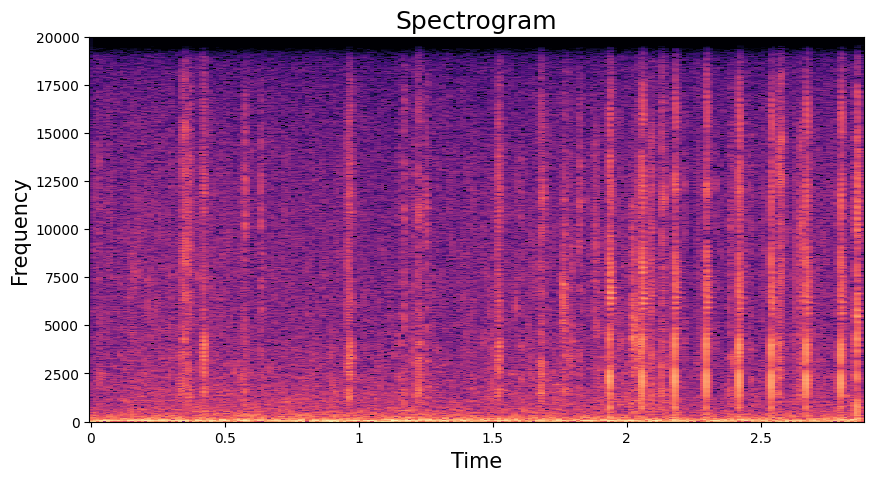


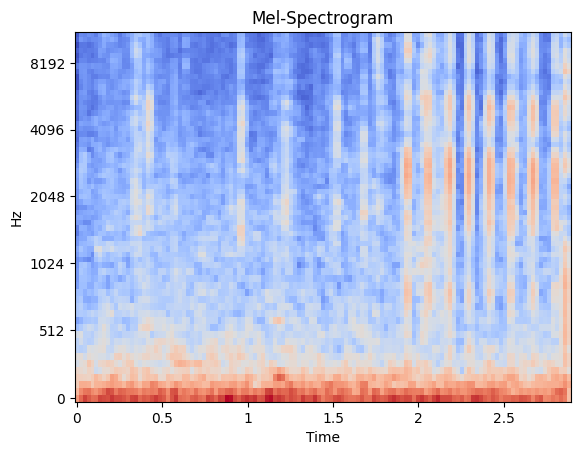


**Sample 12: Bottlenose dolphin**

WMD Sample ID: 94201044


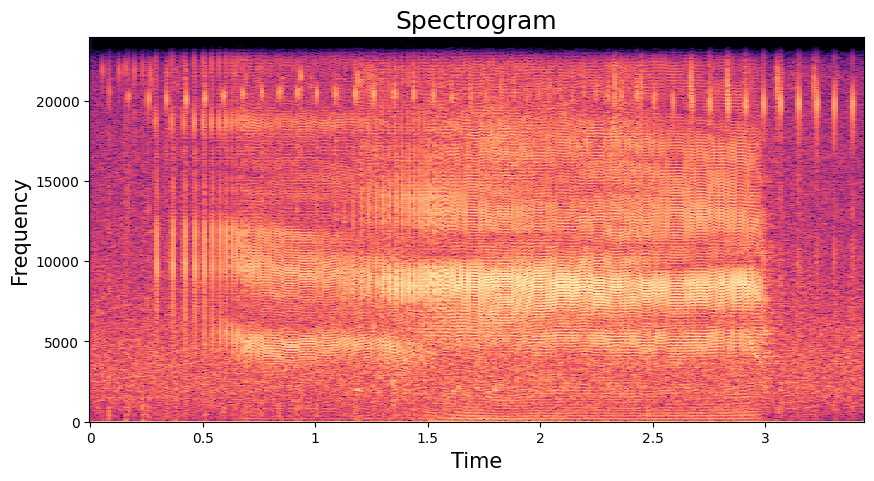

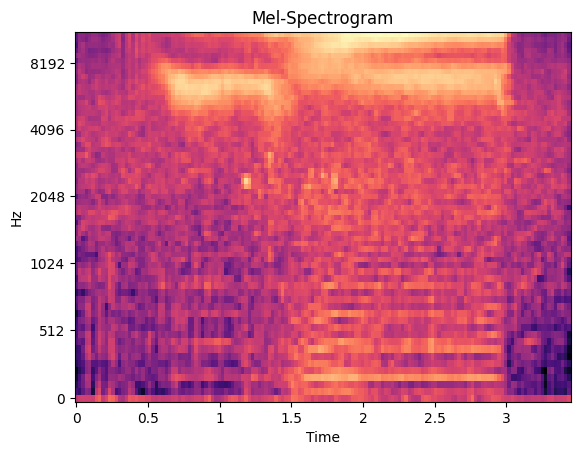


**S1.2: Humpback Whale Vocalizations Spectrograms & Log-Mel Spectrograms**

This section contains six example spectrograms of humpback whale vocalizations from the PBD dataset, and the corresponding Mel-spectrograms used as input in VGGish. The spectrograms and Mel-spectrograms are computed using the librosa python package version 0.10.1 (DOI:10.5281/zenodo.8252662) with n_ftt = 2048 and hop_size = 512.

**Sample 1: July 11 2019 – 3:45:30.000**


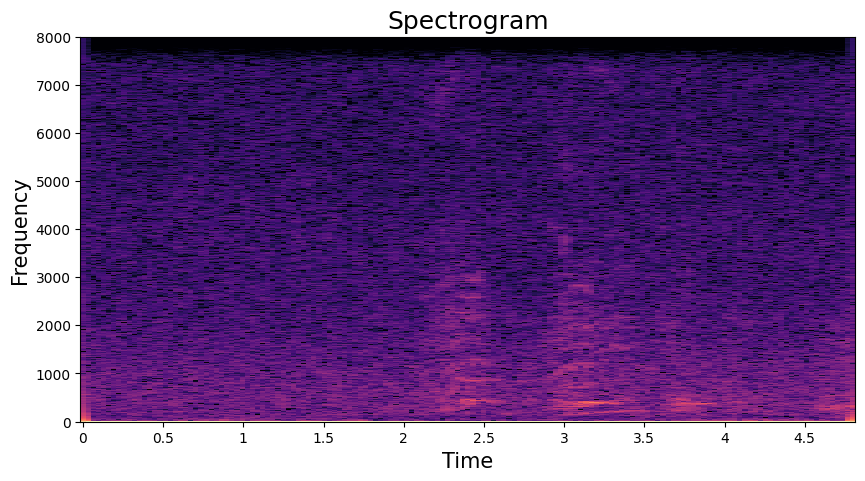


**
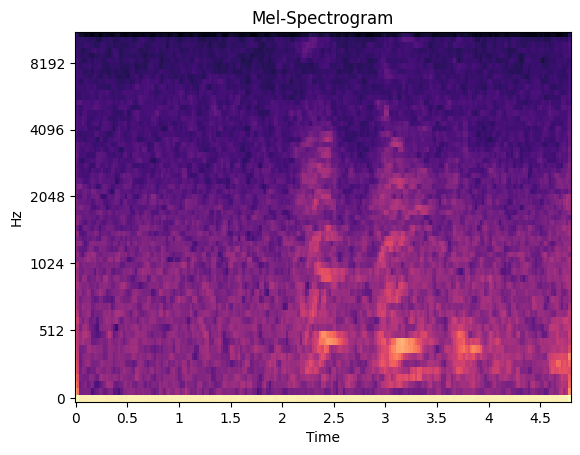
**

**Sample 2: July 11 2019 – 3:45:34.800**

**
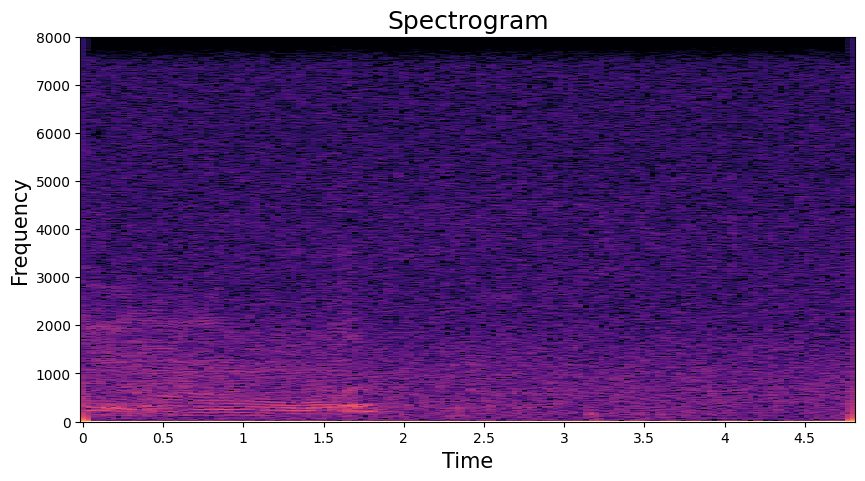
**

**
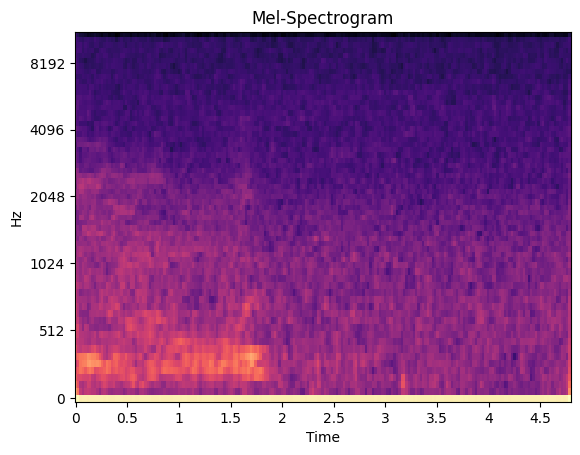
**

**Sample 3: July 17 2019 – 2:47:13.000**

**
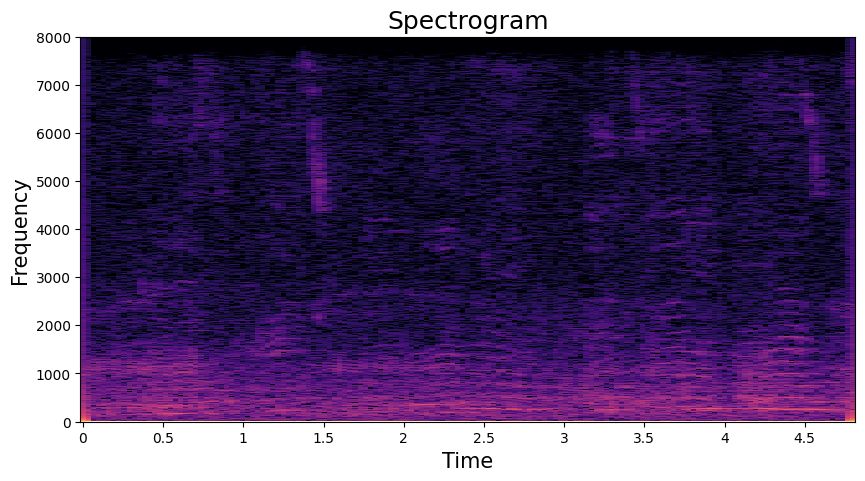
**

**
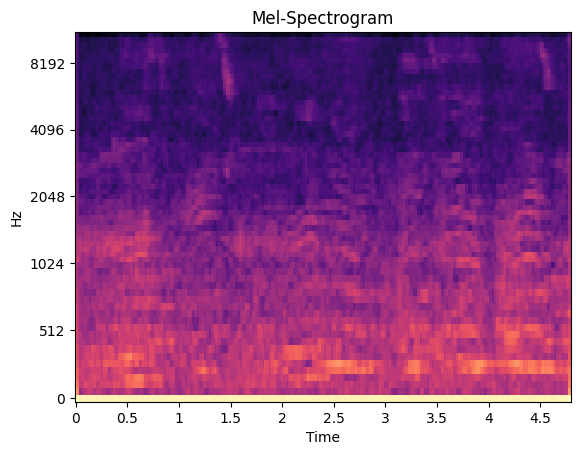
**

**Sample 4: July 17 2019 – 2:47:17.800**

**
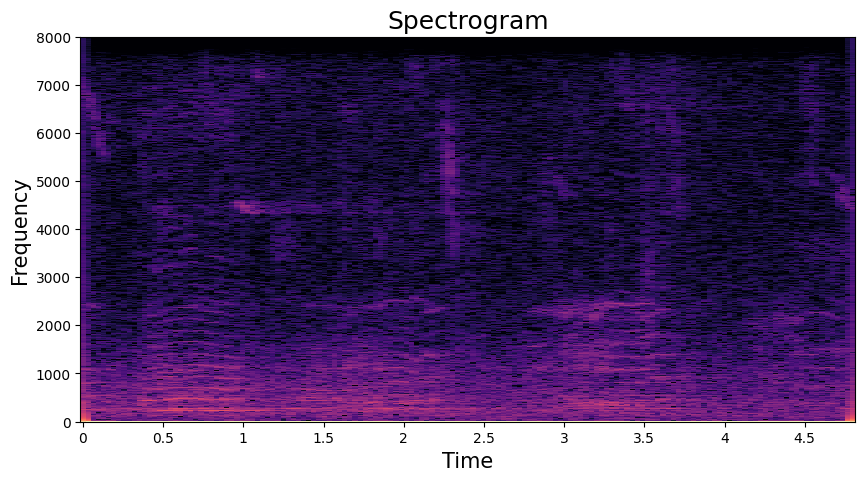
**

**
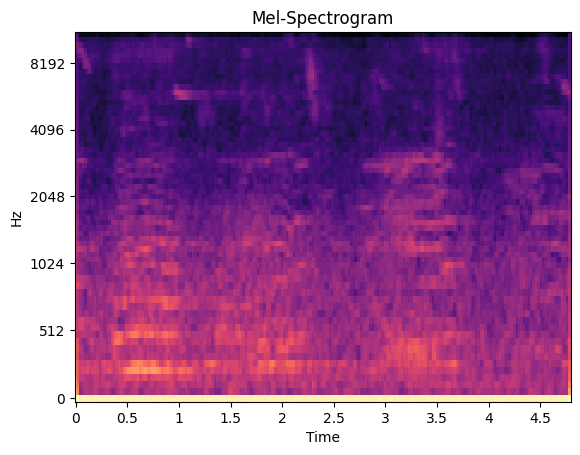
**

**Sample 5: July 21 2019 – 21:11:19.000**

**
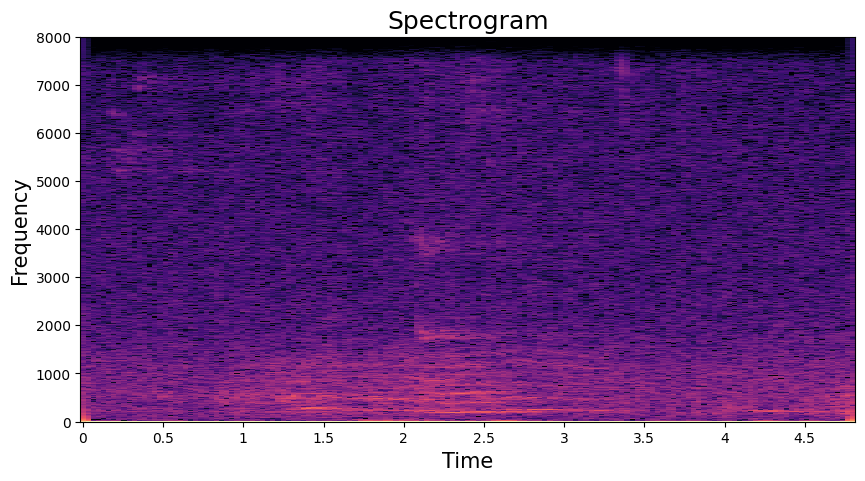
**

**
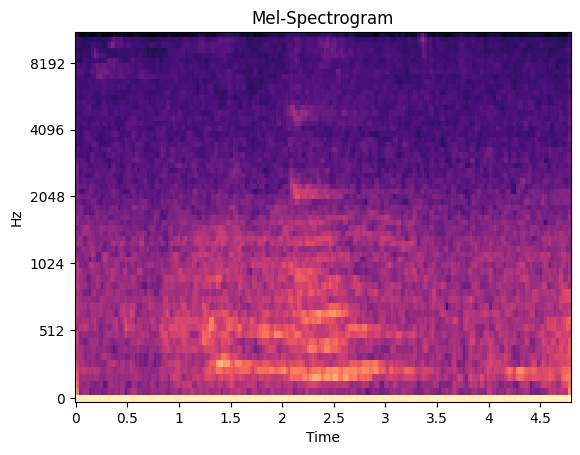
**

**Sample 6: July 21 2019 – 21:11:19.000**

**
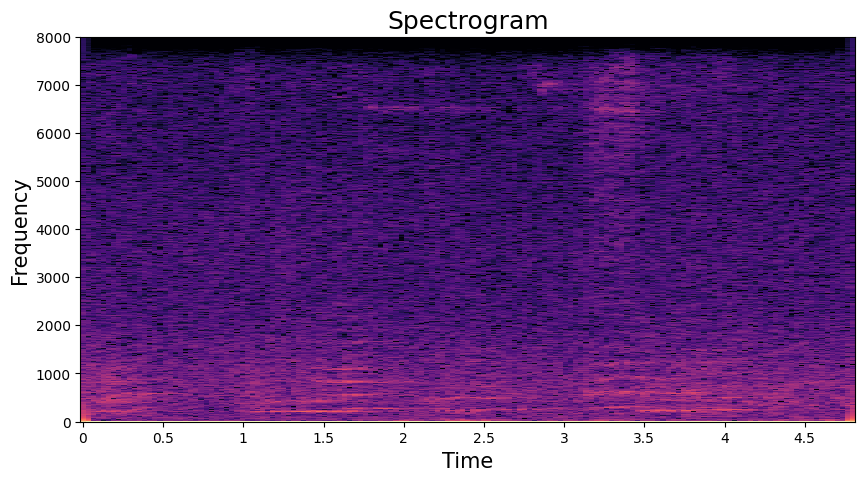
**

**
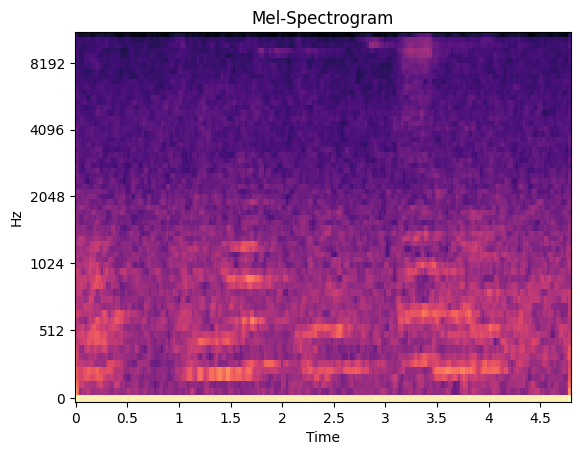
**

**S1.3: August predictions**

This section contains four example spectrograms and Mel-spectrograms of humpback whale detections from the BRF model trained on the PBD dataset: two true positives and two false positives are shown below.

The spectrograms and Mel-spectrograms are computed using the librosa python package version 0.10.1 (DOI:10.5281/zenodo.8252662) with n_ftt = 2048 and hop_size = 512.

**False positive 1: August 2 2019 – 19:13:53.000**
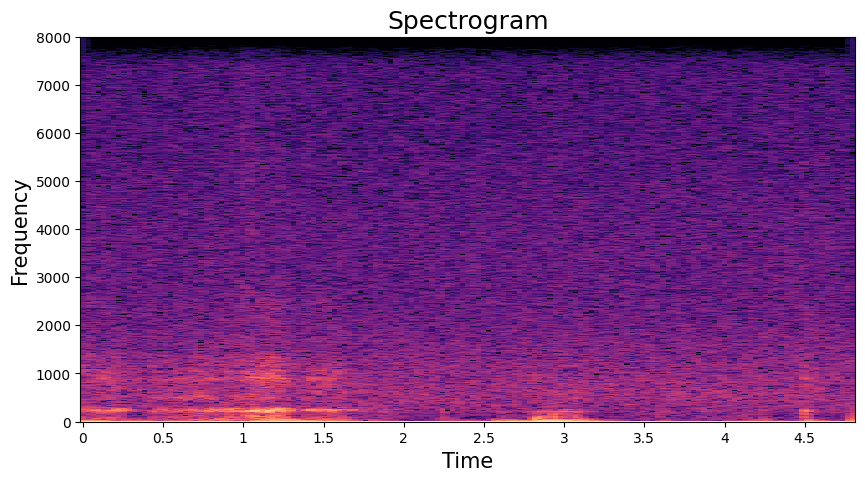


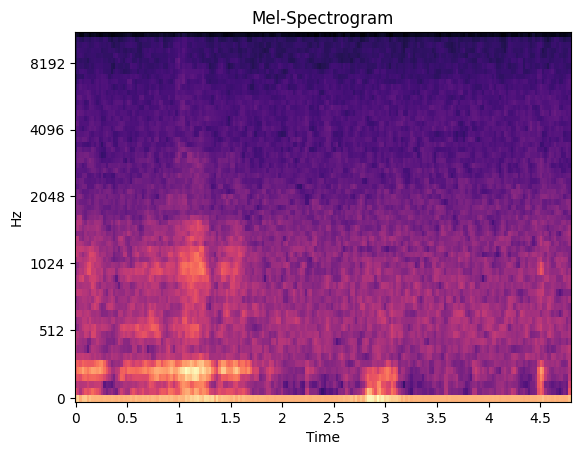


**False positive 2: August 24 2019 – 18:16:58.800**

**
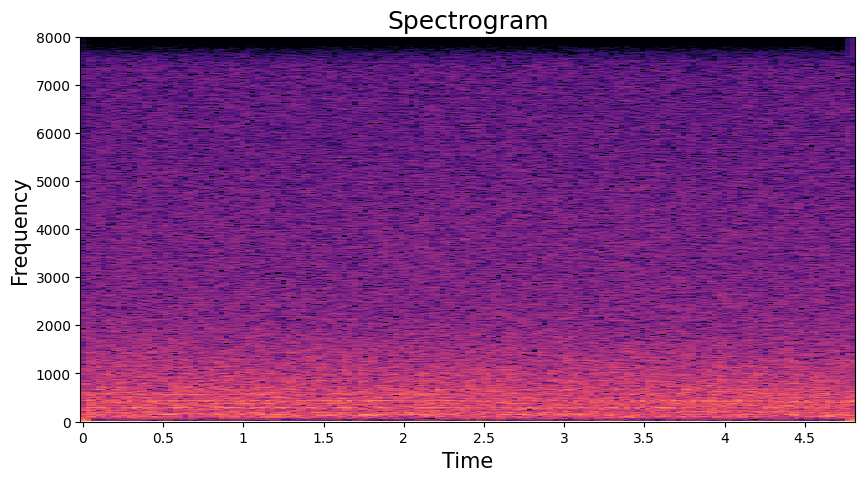

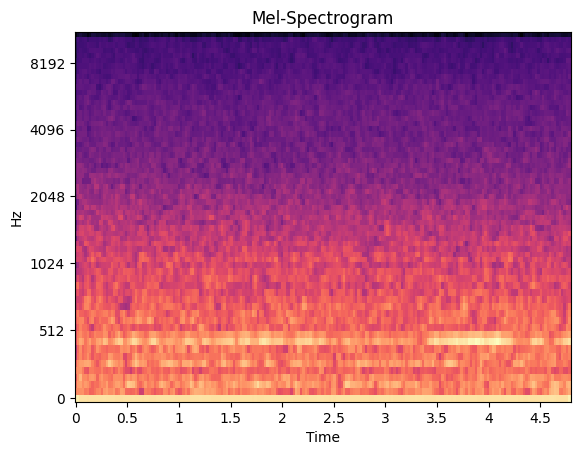
**

**True positive 1: August 23 2019 – 8:41:09.000**

**
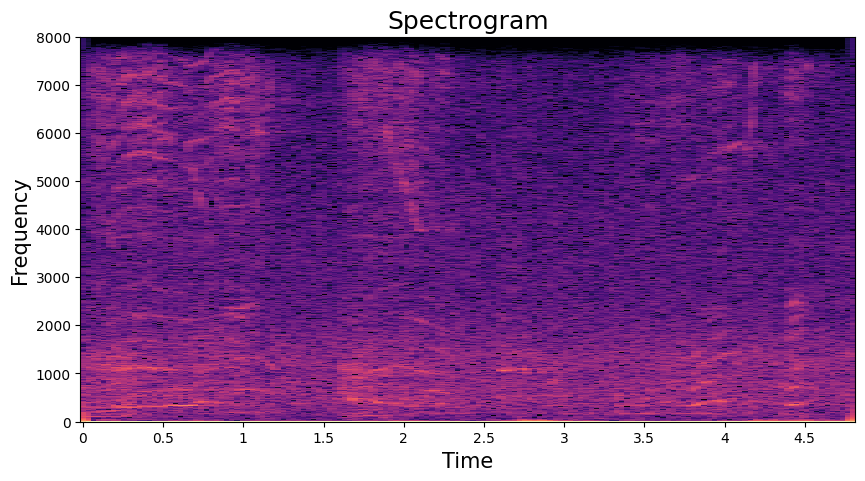
**

**
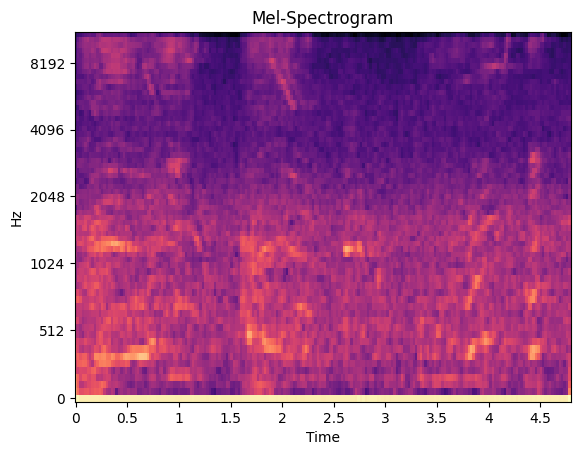
**

**True positive 2: August 23 2019 – 8:41:09.000**

**
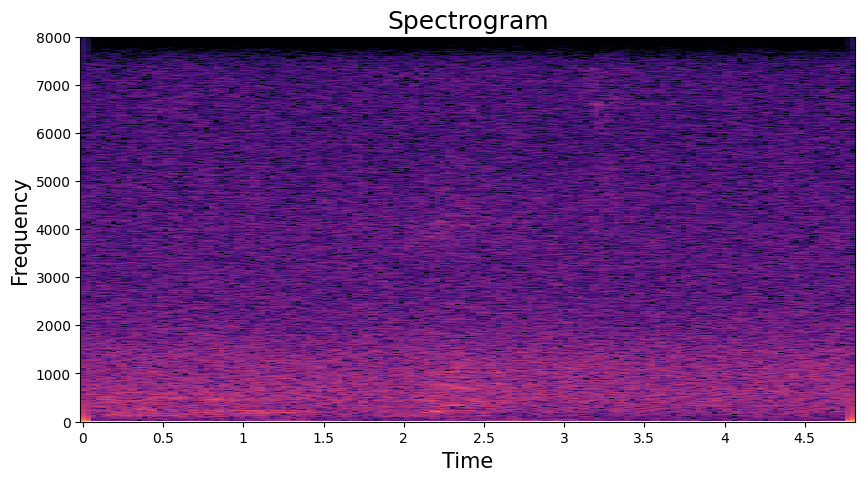
**

**
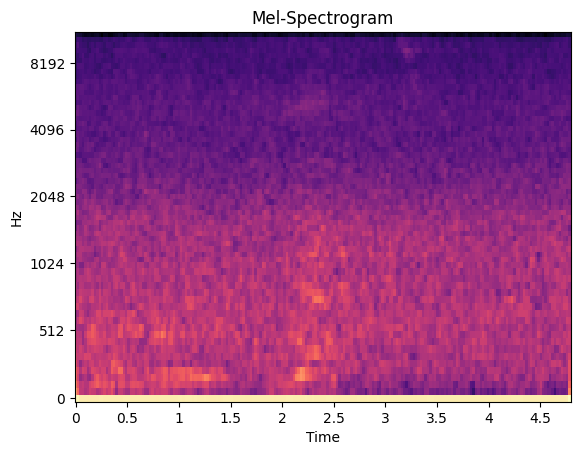
**
