# Supplementary material for: Acoustic features as a tool to visualize and explore marine soundscapes: Applications illustrated using marine mammal passive acoustic monitoring datasets
Source: Ecol Evol. 2024 Feb 21;14(2):e10951. doi: 10.1002/ece3.10951 (PMC10880131; doi:10.1002/ece3.10951)
Supplement: Supplementary file 2 — Appendix S2. [file ECE3-14-e10951-s002.docx]

**APPENDIX S2: Violin Plots, Confusion Matrices, and additional UMAP visualizations.**

**Violin Plots**

The violin plots below compare the two UMAP dimensions generated as embeddings to the acoustic features generated by VGGish for different labels assigned to the acoustic samples from the Watkins Marine Mammal Sounds Database (WMD). The labels include marine mammal taxonomic groups (Fig S2.1), marine mammal species (Fig S2.2), and the locations for humpback and killer whales (Fig S2.3).


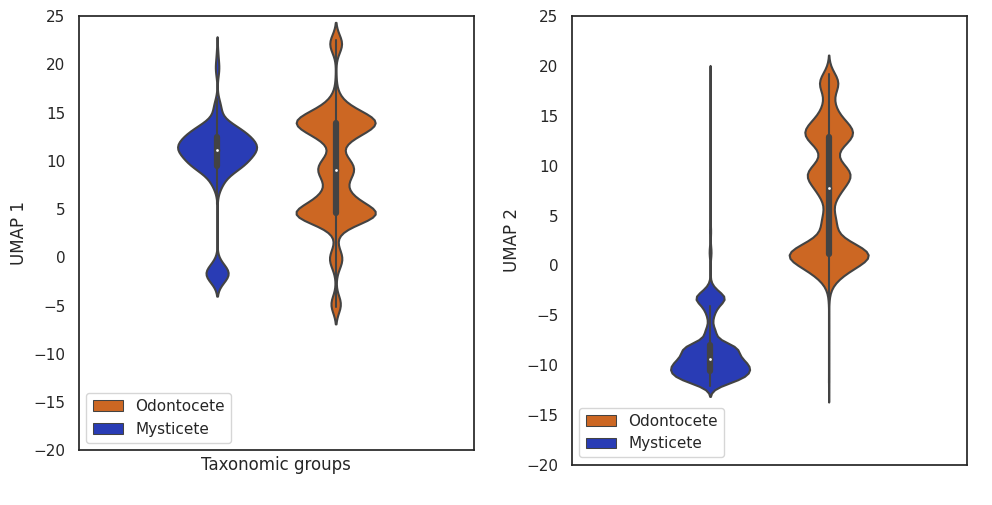


Figure S2.1 Violin plots showing the distribution of UMAP dimensions for the taxonomic group label of the WMD dataset.


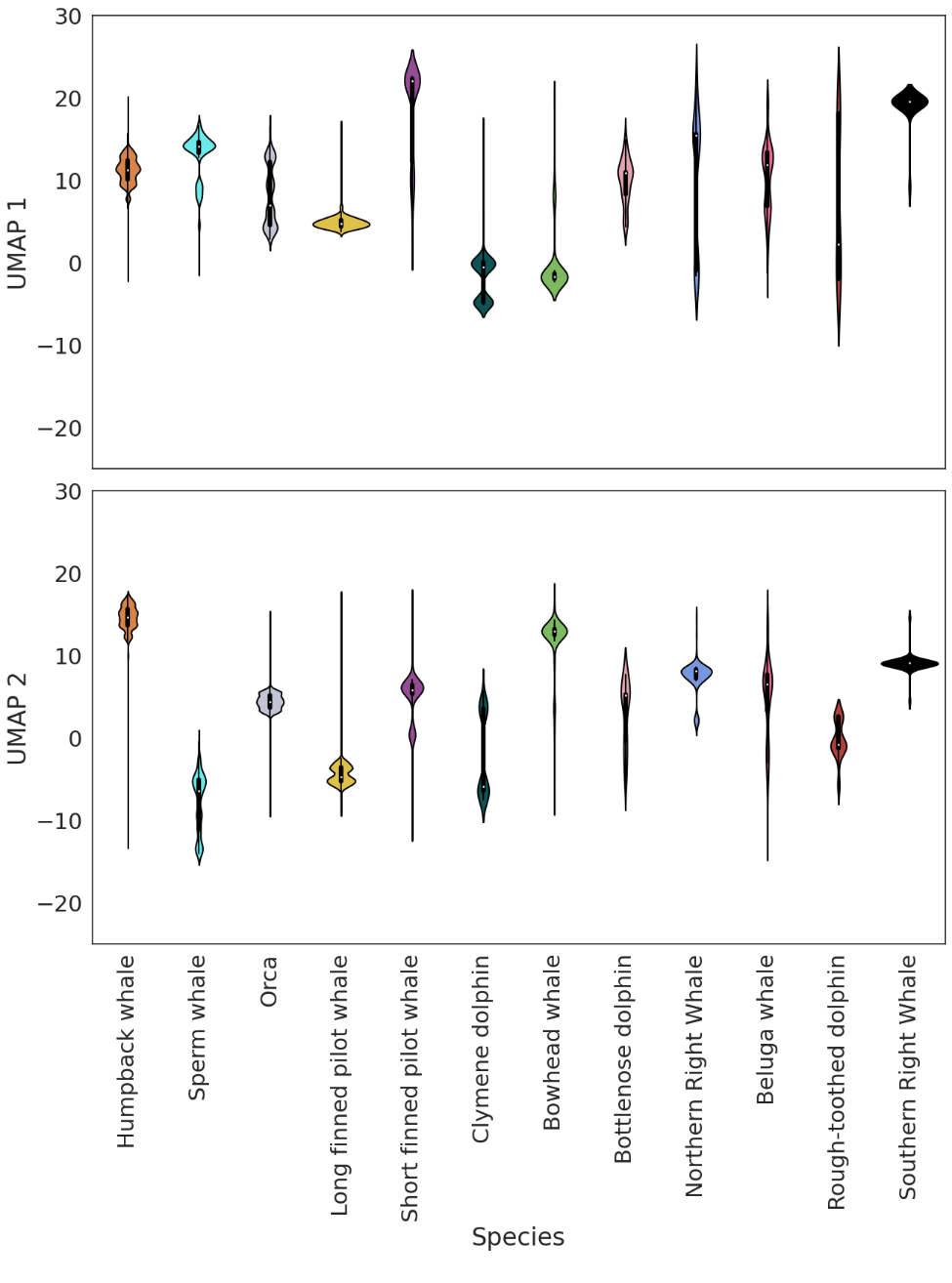


Figure S2.2 Violin plots showing the distribution of UMAP dimensions for the species label of the WMD dataset.


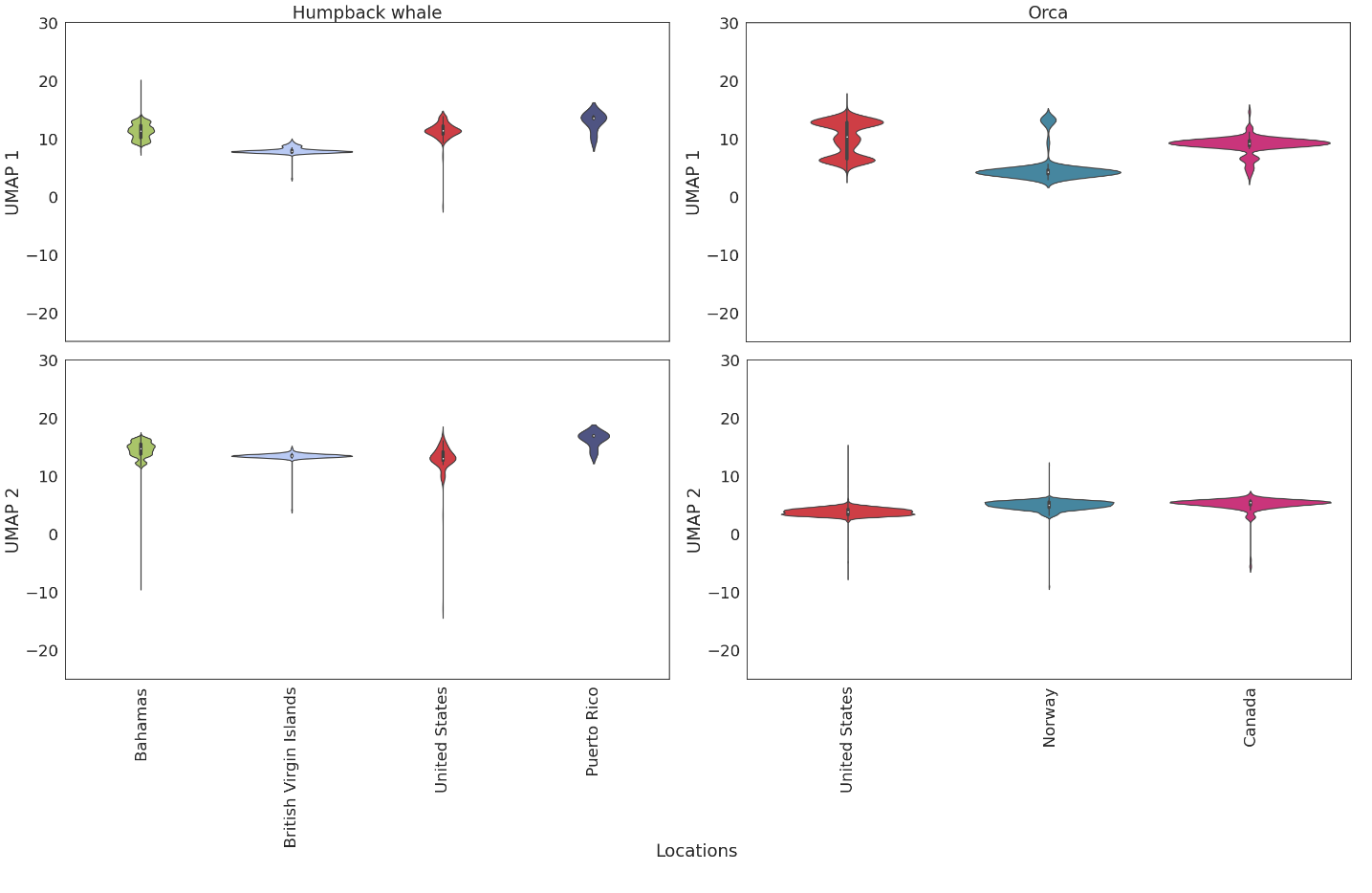


Figure S2.3. Violin plots showing the distribution of UMAP dimensions for humpback whales and orcas according to their sampling location.

**Confusion Matrices**

The confusion matrices reported below report the performance of the balanced random forest classifiers on the testing dataset relative to wind speed (Fig S2.4), surface temperature (Fig S2.5), current speed (Fig S2.6), and presence of humpback whales (Fig S2.7).


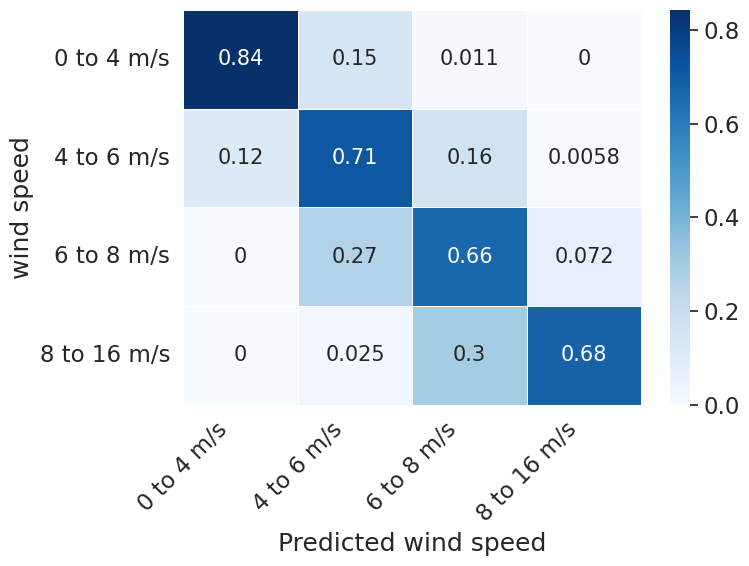


Figure S2.4. Confusion matrix of the testing dataset for the wind speed labels.


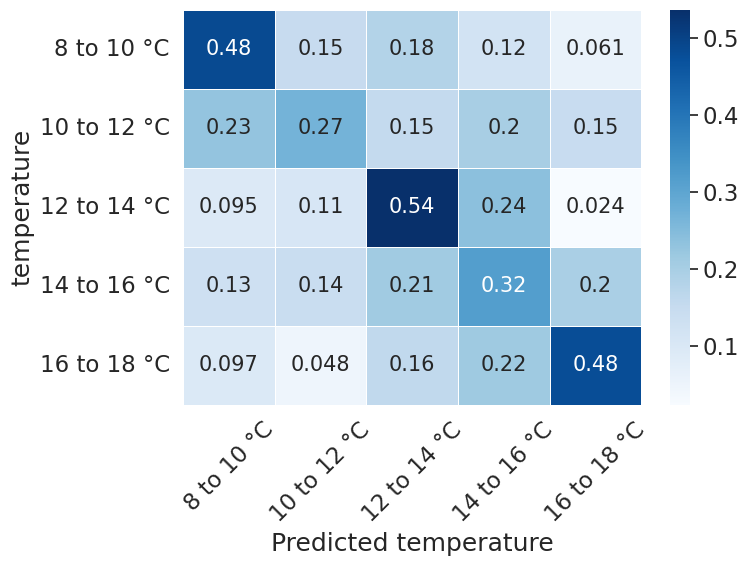


Figure S2.5 Confusion matrix of the testing dataset for the surface temperature labels.


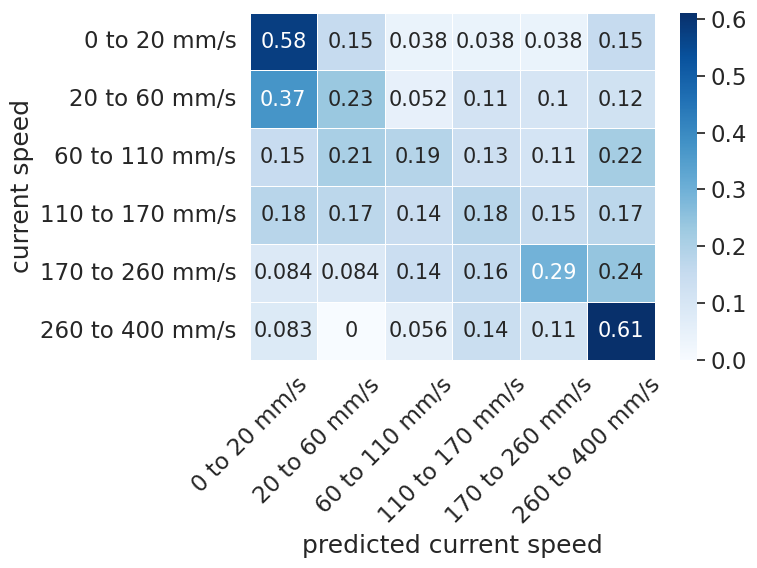


Figure S2.6. Confusion matrix of the testing dataset for the current speed labels.


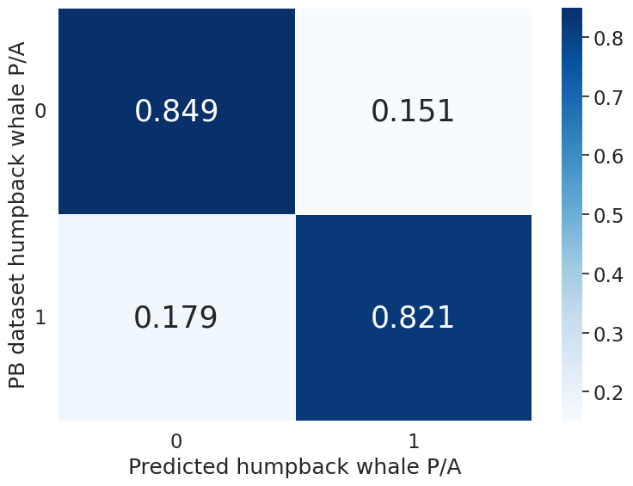


Figure S2.7. Confusion matrix of the testing dataset for the humpback whale presence labels.

**Additional UMAP Visualizations:**

UMAP visualizations for two of the oceanographic variables (Fig 1): surface temperature (Fig S2.8) and current speed (Fig S2.9).


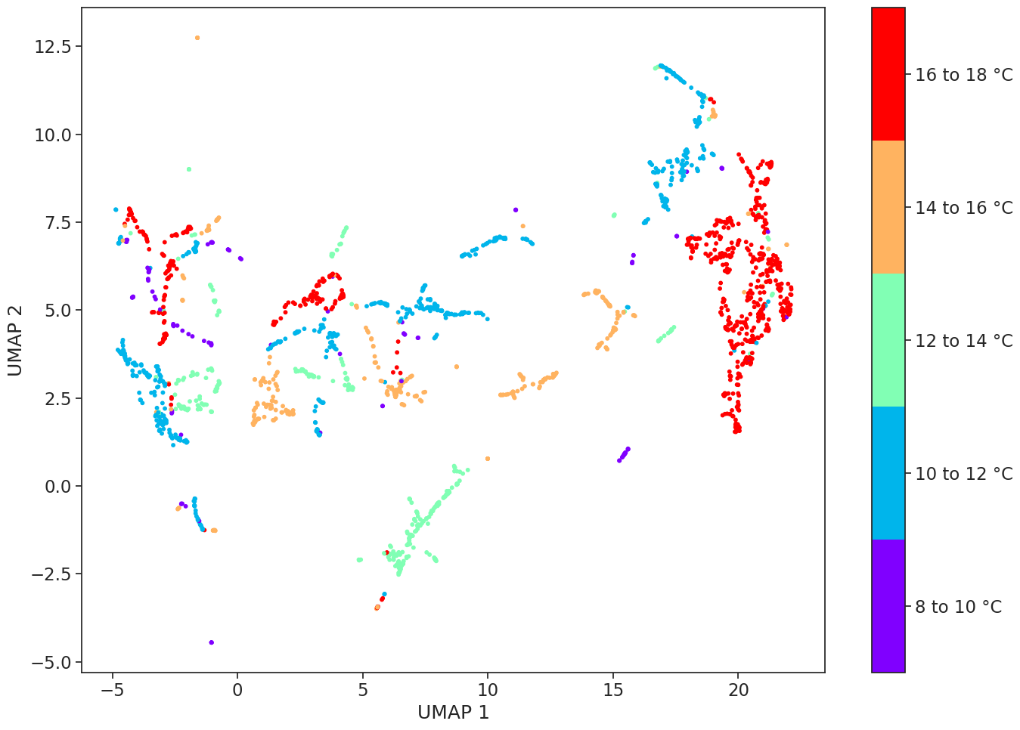


Figure S2.8 UMAP visualization of the surface temperature labels.


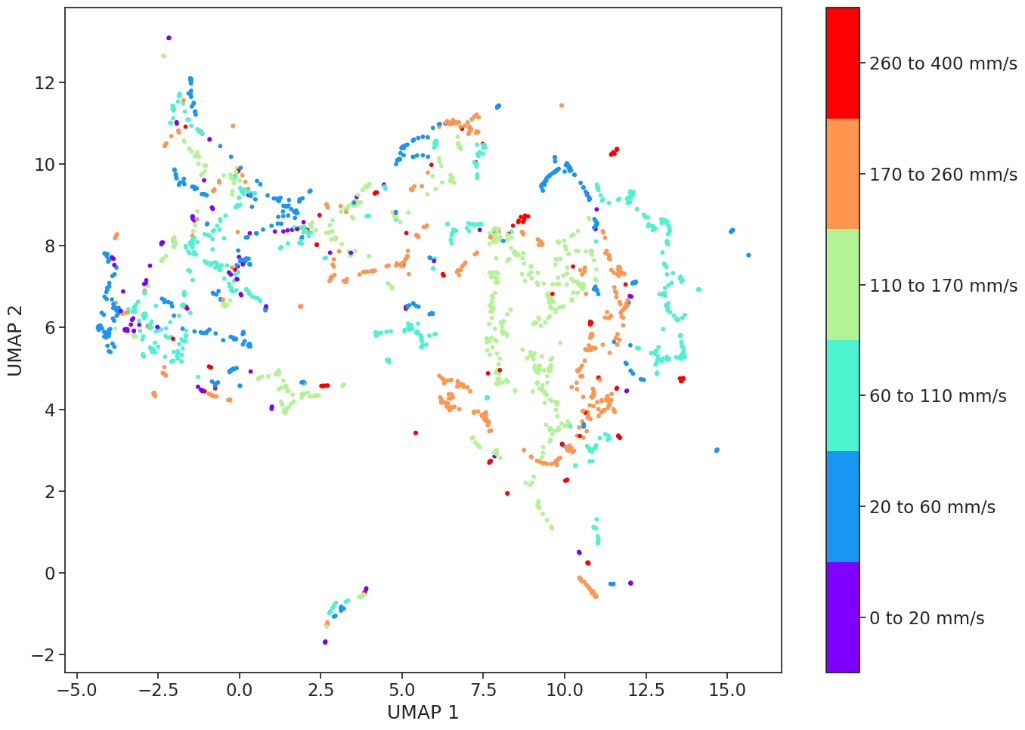


Figure S2.9 UMAP visualization of the current speed labels.
